# Supplementary material for: Modeling Adsorption and Optical Properties for the Design of CO2 Photocatalytic Metal-Organic Frameworks
Source: Molecules. 2021 May 20;26(10):3060. doi: 10.3390/molecules26103060 (PMC8161253; doi:10.3390/molecules26103060)
Supplement: Supplementary file 1 [file molecules-26-03060-s001.zip › molecules-1183007-supplementary.pdf]

Supporting Information

Modeling adsorption and optical properties for the design of CO_2 photocatalytic Metal-Organic Framework

Priscila Chacón [1], Joseelyne G. Hernández-Lima [2] Adán Bazán-Jiménez [3] and Marco A. García-Revilla*

Chemistry Department, Natural and Exact Sciences Division, University of Guanajuato, Noria Alta S/N, 36050-Guanajuato, Mexico.; p.chaconmartinez@ugto.mx (P.C); joseelyne.hernandez@ugto.mx (J.G.H-L); jabazan@ugto.mx (A.B-J); magarcia@ugto.mx (M.A.G-R)

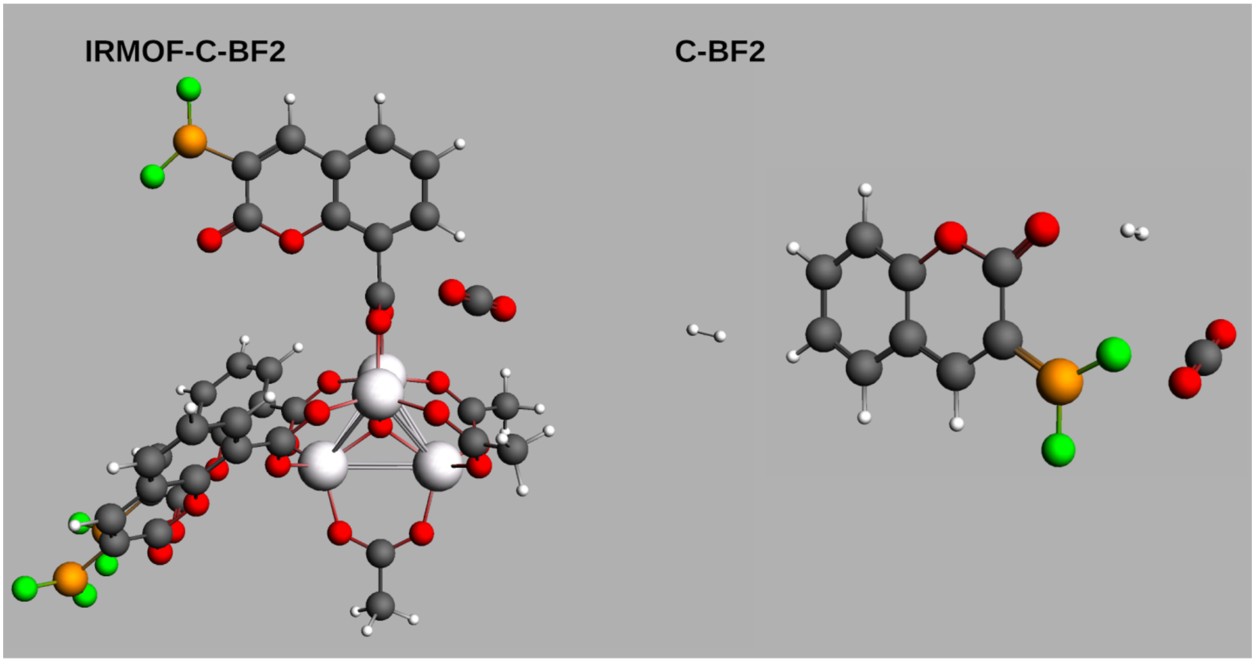

STRUCTURE	GUEST	BOND X-Y*	DISTANCE (Å)
IRMOF-C-BF2	CO2	O – C	4.319
	CO2	O – C	3.867
	CO2 + H2	C – H**	4.971
C-BF2	H2	H – O	2.650
	CO2 + H2	O – H*	3.455
	CO2	C – O**	1.171
	H2	H – H**	0.756

* In bond column X represents guest atom and Y structure atom. ** Both atoms belong to the guest(s) molecule(s) indicated.

Figure SI-1. Optimized geometries for the calculation of the brightest excited states of the primitive cell (IRMOF-C-BF_2) and the linker (C-BF_2) in the presence of H_2 and CO_2. The distances specified are shown in Angstroms (Å). Notice the small distances between the atoms of the guests and boron of BF_2 substituents and the O atom of the carbonyl moiety. The association of one oxygen of CO_2 to the boron can be explained with an acid-base chemistry according to Lewis, in which the former behaves as a base and the later as an acid. A similar explanation can be given for H_2 association with the carbonyl group. In this way, the frustrated Lewis pair can be of assistance to the absorption of the guest molecules.

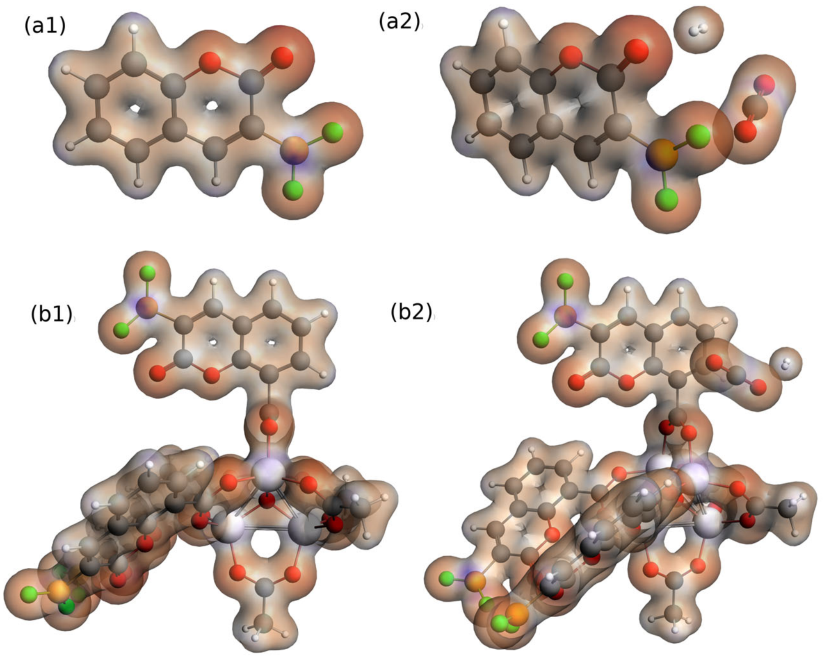

Figure SI-2. Molecular electrostatic properties of C-BF_2 linker and IRMOF-C-BF_2:

Isosurfaces of density and potential of the Self Consistent Field for C-BF_2 (a1, a2) and IRMOF-C-BF_2 (b1, b2) in the presence of H_2 and CO_2 as guest molecules. The considered value for those isosurfaces is settled 0.003. The most electron-deficient places are the boron atoms (a1, b1) for both the linker and MOF. The most electron-rich region in absence of the metallic cluster is the carbonyl oxygen (a1). Nevertheless, the most electron-rich region is not the same in the case of IRMOF-C-BF_2 (b1); here the oxygen atoms of the OCO bridges display this characteristic. For this reason, we can assume that CO_2 oxygen atoms would adsorb near the boron atoms. Although this is observed for C-BF_2, it is otherwise for the IRMOF-C-BF_2, see Figure SI-1. In the latter, the absorption of the electron-deficient carbon of CO_2 near the most electron-rich moiety of IRMOF-C-BF_2 becomes more relevant than the adsorption directed by oxygen atoms of CO_2 to the boron atoms of ligand. Comparing (a1) and (a2) Molecular Electrostatic Potentials (MEPs), there is no change in the electron density neither in C-BF_2 nor in the guests during adsorption. Considering that the distance among the guests and IRMOF-C-BF_2 is larger than for C-BF_2, it is reasonable that the MEPs do not show important changes from IRMOF-C-BF_2 isolated (b1) compared with the configuration in which the absorption has taken place (b2).

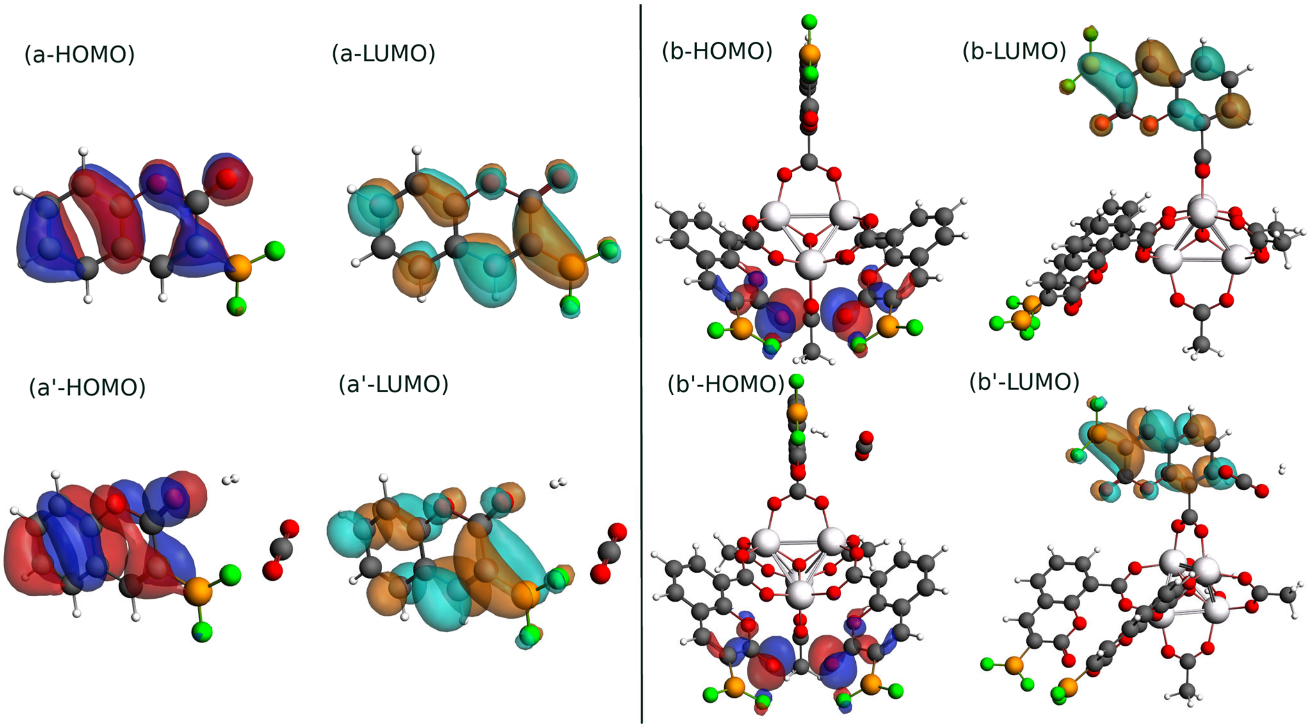

Figure SI-3. Frontier Orbitals of C-BF_2 and IRMOF-C-BF_2. Geometry of the Highest Occupied Molecular Orbital (HOMO) and Lowest Unoccupied Molecular Orbital (LUMO) of C-BF_2 and IRMOF-C-BF_2, before and after the absorption of H_2 and CO_2 as guest molecules. The images that include guest molecules are indicated with an apostrophe (ʹ) after the letter used to distinguish between C-BF_2 (a) and IRMOF-C-BF_2 (b). Regarding C-BF_2, both frontier orbitals keep their geometry and location over the linker's structure comparing the isolated linker (a) and the structure of the adsorbed guests in the linker (a'). The HOMO has a bonding character mostly on the coumarin-based-rings (eight lobes above and below the plane of symmetry of the molecule, six of them above and below the rings and two on the oxygen atom of the carbonyl moiety), whereas the LUMO shows an antibonding character (fourteen lobes, above and below the plane of symmetry of the molecule). In addition, the apparent bonding character across the bond-path (see QTAIM results) that links the boron atom and the carbon of the carbonyl group in the LUMO support the region near the boron atom as the most prone to interact with the guests' HOMO. For the case of IRMOF-C-BF_2 (b and b'), HOMO and LUMO are located on the linkers, excluding the MOF's central cluster as the probable absorption moiety. Such situation corresponds to the observed profile of the MEP: the electron density available for absorption is not in the metallic cluster of the MOF. The HOMO shows mainly antibonding character (sixteen lobes); it is distributed over two linkers, it has a symmetry plane that includes the coordinate axis that goes through the coumarin moiety whose atoms do not contribute to the orbital, this results in a symmetrical shape of the lobes in both linkers involved. The LUMO has antibonding character, with its highest probability zones above and below the plane of one of the linkers, a similar distribution to the observed for C-BF_2; in a similar fashion as C-BF_2, the orbital's morphology is kept when the guest are absorbed (b').

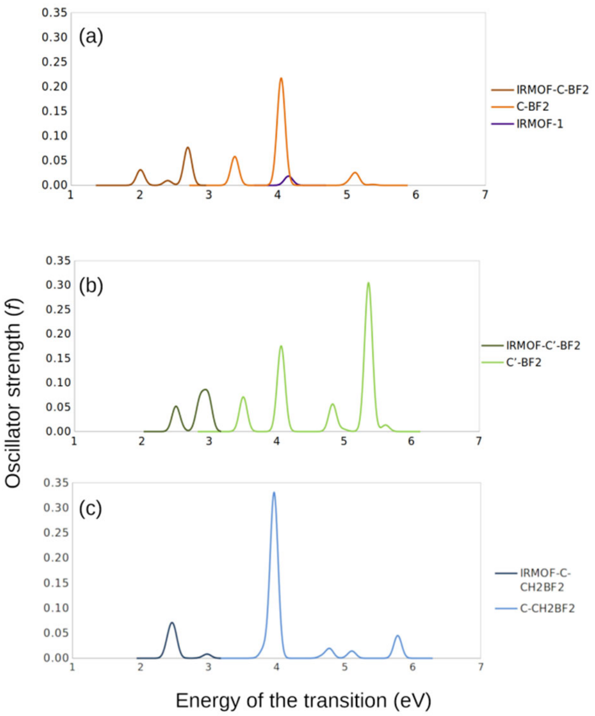

Figure SI-4. Calculated UV and Visible spectra of MOF and its parent linkers in the presence of H_2 and CO_2 as guest molecules. (a) IRMOF-C-BF_2, C-BF_2 and IRMOF-1; (b) IRMOF-C'-BF_2 and C'-BF_2; (c) IRMOF-C-CH_2BF_2 and C-CH_2BF_2. The oscillator strength is plotted as a function of the transition's energy (eV). The *f* value of IRMOF-C-BF_2 and IRMOF-1 is multiplied by a factor of 10.

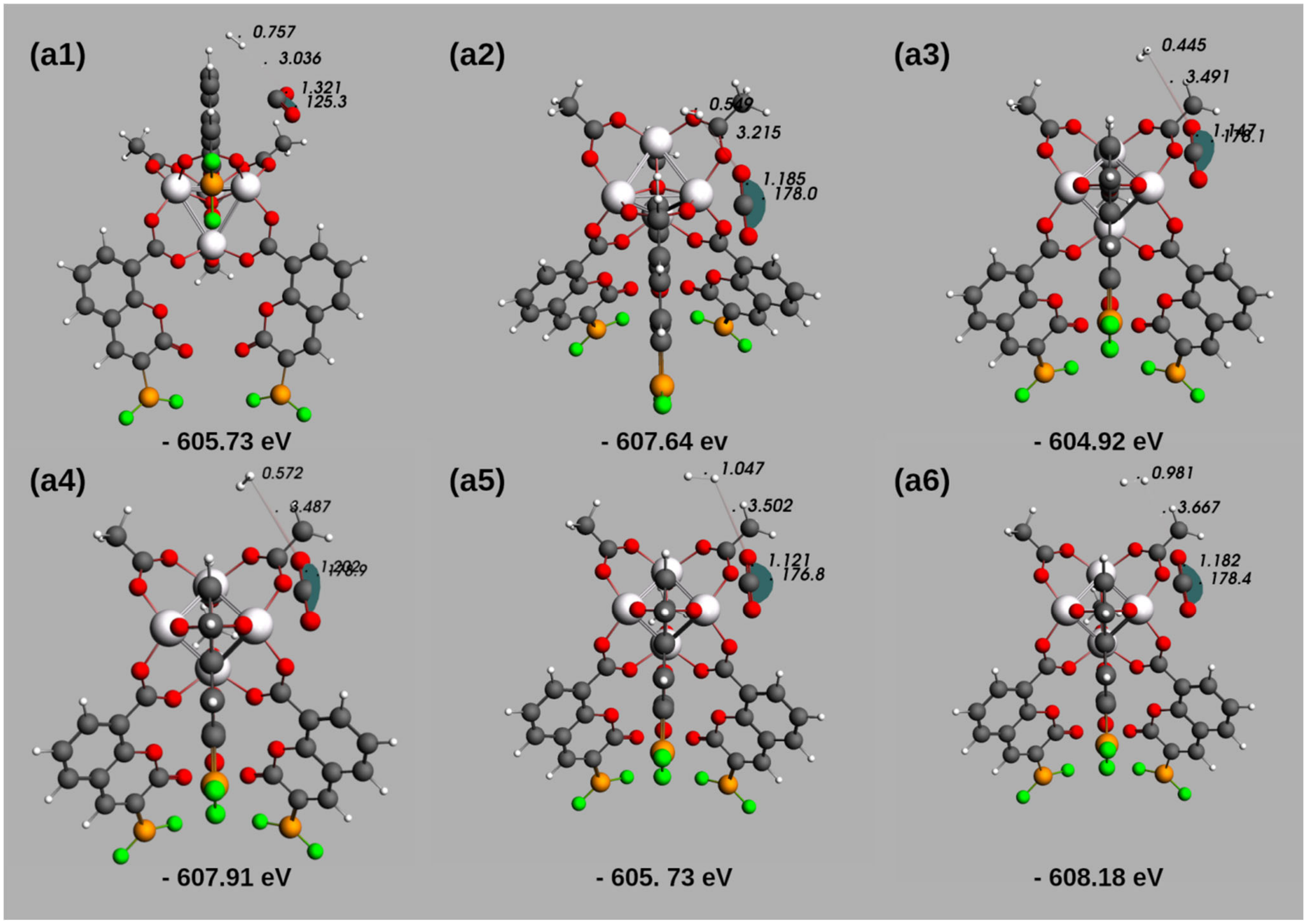

Figure SI-5. Energetic maximums obtained during excited states optimizations for IRMOF-C-BF_2-H_2-CO_2. The energies and the most significant distances and bond angles of all geometries are included. As a comparison, the energy of the minimum is – 608.807 eV. The first maximum (a1) shows appreciable distortion to the OCO angle in CO_2 (30.39% of distortion, 125.3º) and elongation of the OC distance in 13.15% (1.321 Å); H2 is placed 3.036 Å away from CO2 with no appreciable HH length distortion. The next two energetic maximums (a2, a3) present just slight elongation in one of the OC distances of CO_2 (lower than 2.5% of the equilibrium distance for isolated CO_2); HH distance is decreased in both cases (to 0.549 Å or 26.90% of decrease for a2, and 0.455 Å or 39.41% of decrease for a3); however, the distance among the guest molecules increases in some extent (to 3.215 Å for a2, and to 3.491 Å for a3). In the named (a4) maximum, compression of HH bond length (to 0.572 Å or 23.83 % of decrease) and elongation of OC distance in CO_2 (to 1.202 Å or 2.38% of decrease) can be perceived; the distance among both guests is 3.487 Å. In the fifth maximum (a5), the guest molecules are 3.502 Å apart; HH and OC bond lengths are elongated (to 1.047 Å or 39.41 % of elongation, and to 1.121 Å or 4.51% of elongation, respectively). The last energetic maximum (a6) is just slightly different from the previous: the guest molecules are 3.667 Å apart; and HH bond length is elongated (to 0.981 Å or 30.63 % of elongation). The energetic differences among all maximums are lower than 2.72 eV.

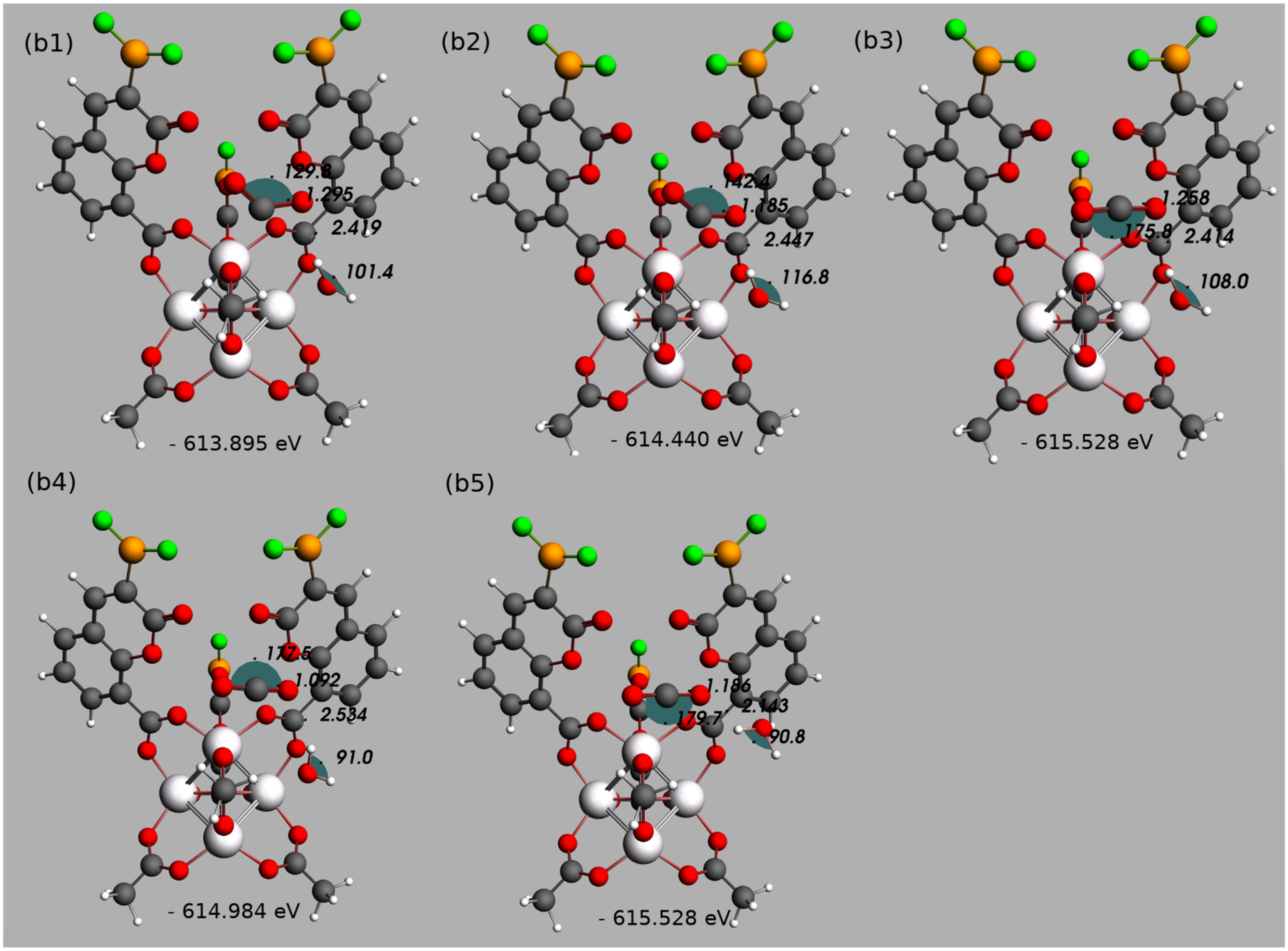

Figure SI-6. Energetic maximums obtained during excited states optimizations for IRMOF-C-BF_2-CO_2-H_2O. The energies and the most significant distances and bond angles of all geometries are included. As a comparison, the energy of the minimum is – 616.263 eV. IRMOF-C-BF2 keeps guests absorbed while the distance among the guests falls in the range of 2.143 Å to 2.534 Å. OCO angle is distorted in the first two maximums (to 129.3° or 28.17% of distortion for b1, and 142. 4° or 20.89% of distortion for b2), but kept almost unchanged in the next three. In the first maximum (b1), only one OC bond length is elongated (to 1.295 Å or 10.31% of elongation); HOH angle in water is slightly reduced (to 101.4° or in 2.97%) with no distortion of bond lengths. HOH angle in H_2O opens in the next maximum (b2) (to 116.8° or 11.77% of distortion) with no appreciable modification to the equilibrium bond lengths for both guests. The third maximum (b3) shows a longer OC distance (1.258 Å, or 7.16% of elongation) than the equilibrium one for CO_2; HOH angle in H_2O is also distorted (108.0° or 3.35% of distortion). The last two maximums (b4 and b5) are characterized by a compressed HOH angle in the water guest (91.0° or 12.92% of compression for b4, and 90.8° or 13.11% of compression for b5); while both OH bond distances in water are kept constant, OC bond lengths are distorted for CO_2 (1.092 Å or 6.99% of distortion for b4, and 1.186 Å or 1.02% of distortion for b5). The energetic differences among all maximums are lower than 1.36 eV.

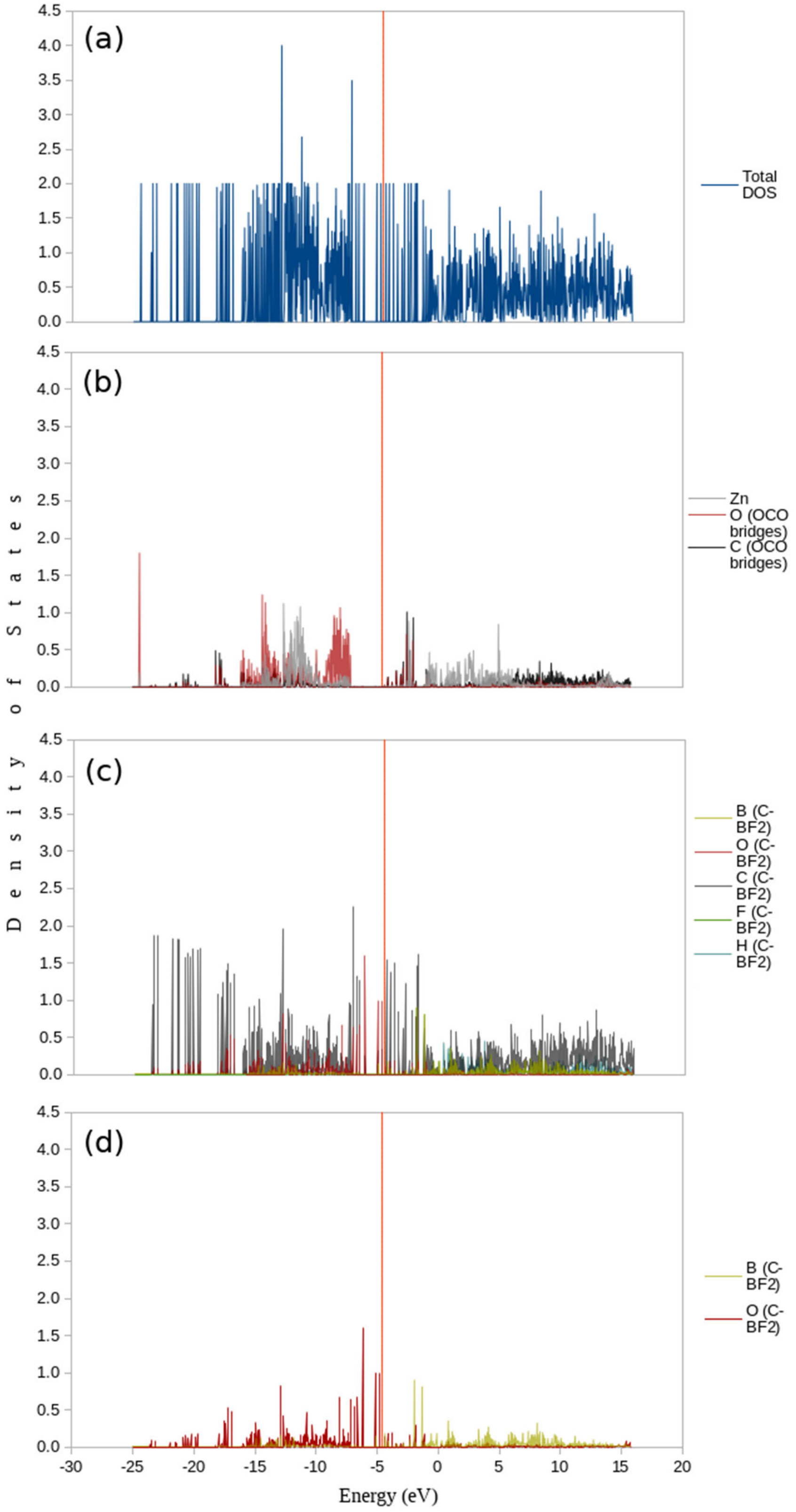

(a)
Total DOS
(b)
Zn
O (OCO bridges)
C (OCO bridges)
(c)
B (C-BF2)
O (C-BF2)
C (C-BF2)
F (C-BF2)
H (C-BF2)
(d)
B (C-BF2)
O (C-BF2)
Density of States
Energy (eV)
4.5
4.0
3.5
3.0
2.5
2.0
1.5
1.0
0.5
0.0
-30
-25
-20
-15
-10
-5
0
5
10
15
20

Figure SI-7. Density of States of IRMOF-C-BF_2, as a function of energy (eV). The DOS are represented as (a) Total Density of States, TDOS, and Partial Density of States for the following fragments: (b) Central cluster, (c) C-BF_2 linker and (d) FLPs in linkers. The orange line present in all charts indicate the Fermi Level, with a value of – 4.63 eV.

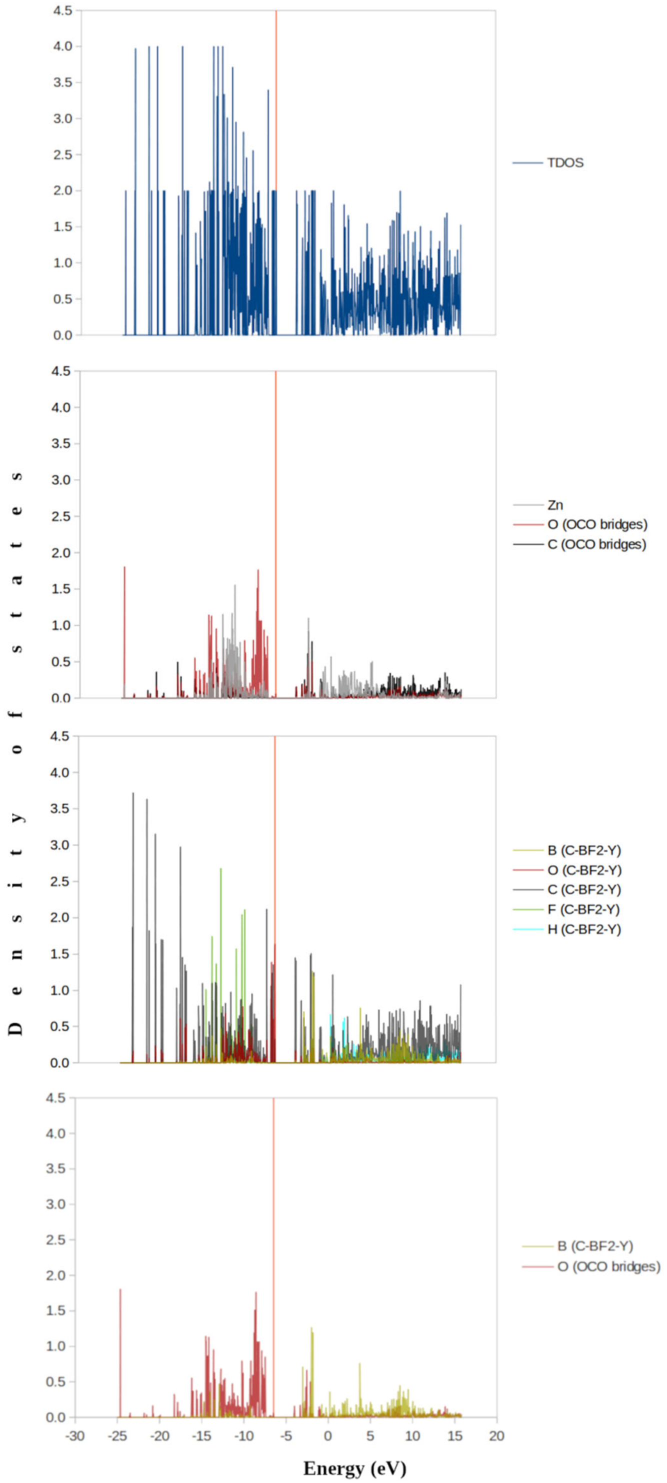

Figure SI-8. Density of States of IRMOF-C'-BF_2, as a function of energy (eV). The DOS are represented as (a) Total Density of States, TDOS, and Partial Density of States for the following fragments: (b) Central cluster, (c) C'-BF_2 linker and (d) FLPs formed between the O atoms of the OCO bridges and the -BF_2 groups on the linker to which they are attached. The orange line present in all charts indicate the Fermi Level, with a value of – 4.63 eV.

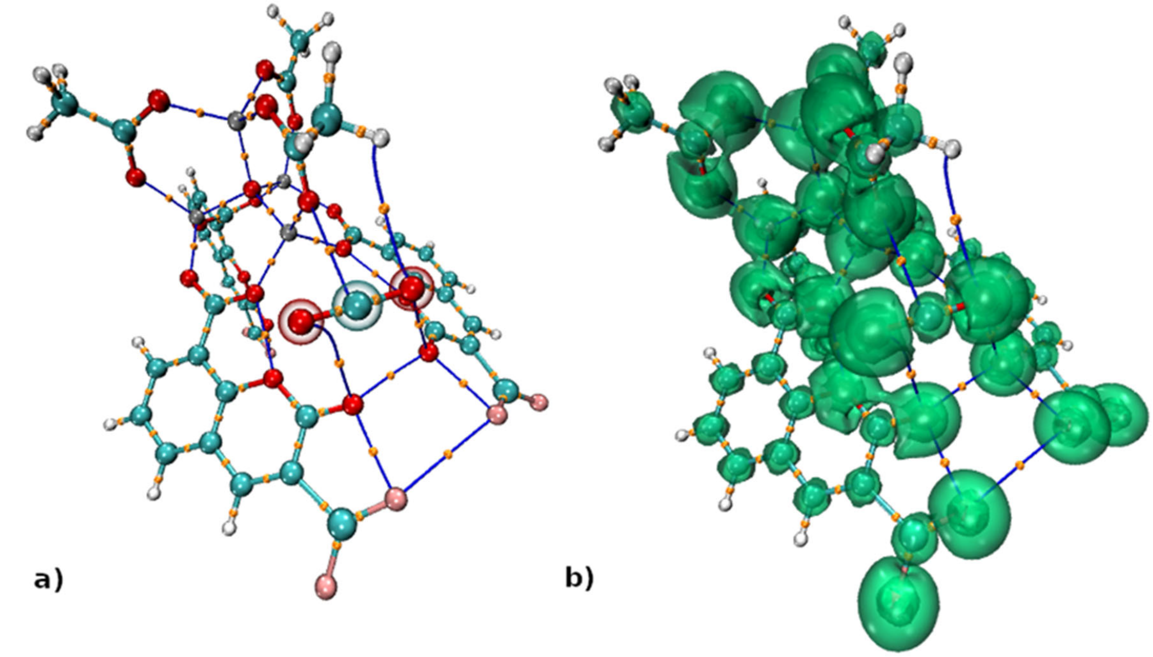

Figure SI-9. a) Electron density topologies for IRMOF-C-BF_2···CO_2. Bond critical points (BCP) are shown in orange, paths are shown in blue. b) Laplacian of electron density isosurface in green (isovalue 0.3 a.u.).

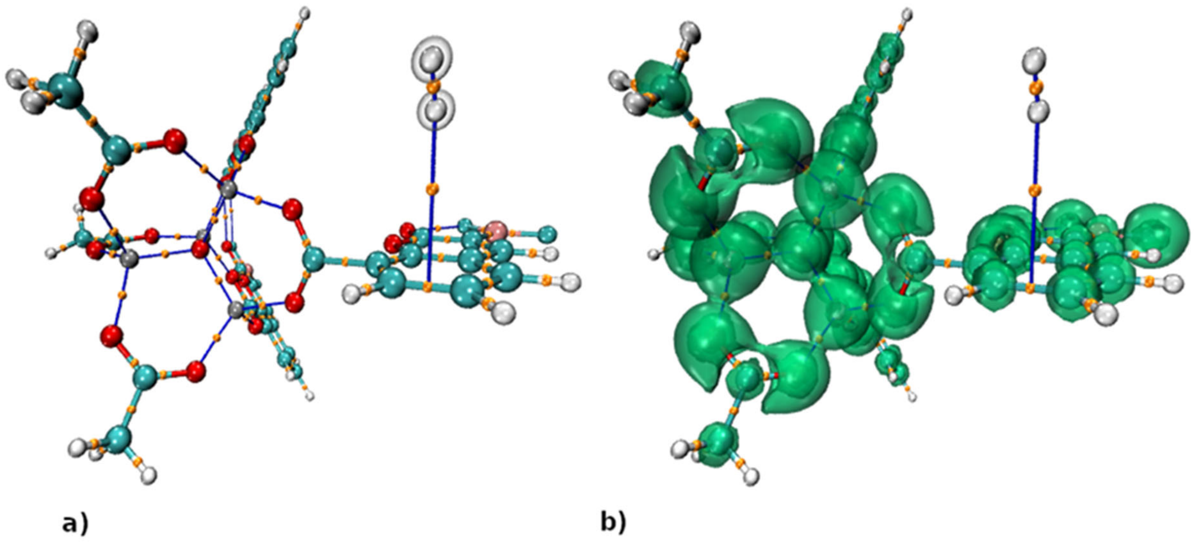

Figure SI-10. a) Electron density topologies for IRMOF-C-BF_2···H_2. Bond critical points (BCP) are shown in orange, paths are shown in blue. b) Laplacian of electron density isosurface in green (isovalue 0.3 a.u.).

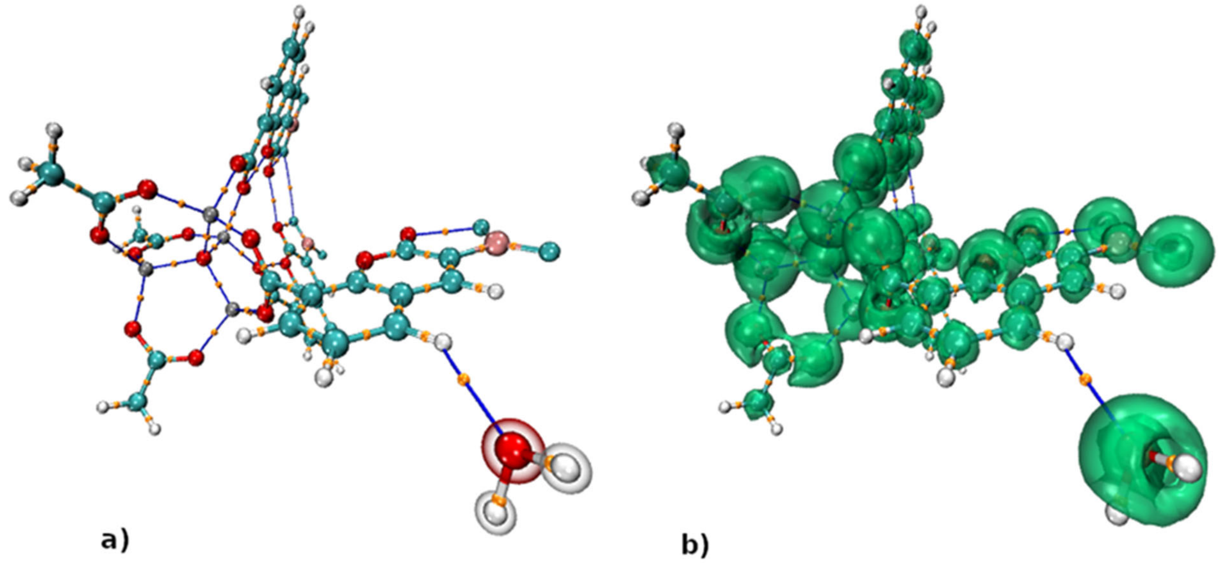

Figure SI-11. a) Electron density topologies for IRMOF-C-BF$_2$···H$_2$O. Bond critical points (BCP) are shown in orange, paths are shown in blue. b) Laplacian of electron density isosurface in green (isovalue 0.3 a.u.).

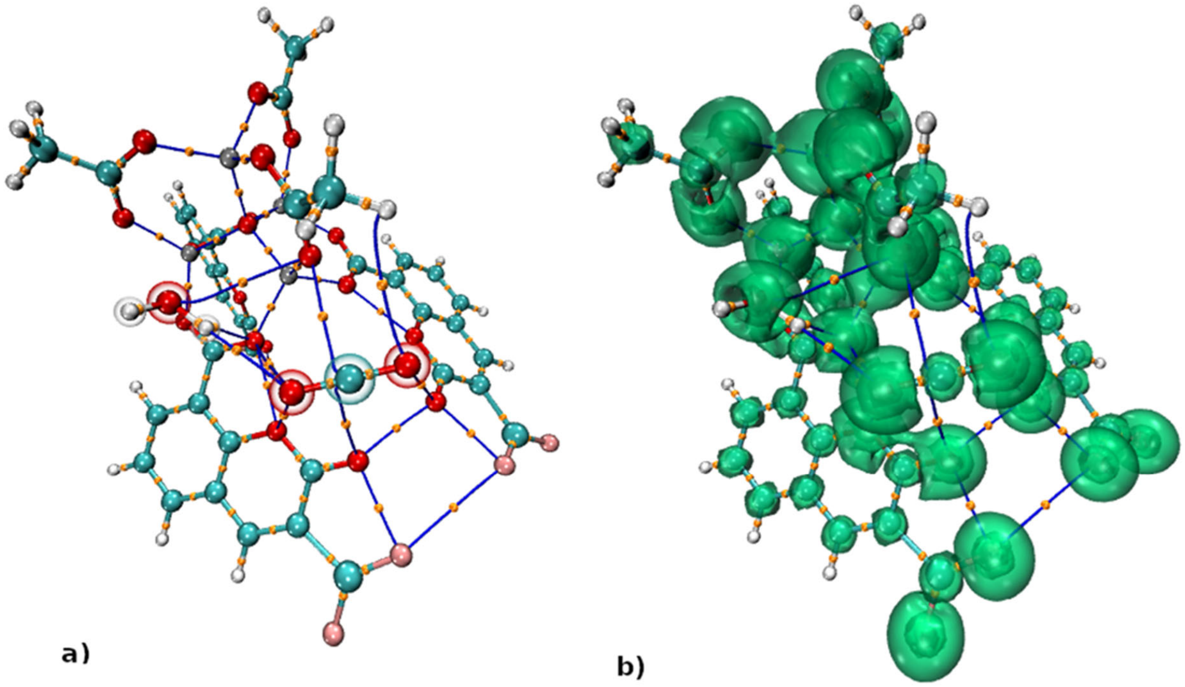

Figure SI-12. a) Electron density topologies for IRMOF-C-BF$_2$---CO$_2$ H$_2$O. Bond critical points (BCP) are shown in orange, paths are shown in blue. b) Laplacian of electron density isosurface in green (isovalue 0.3 a.u.).

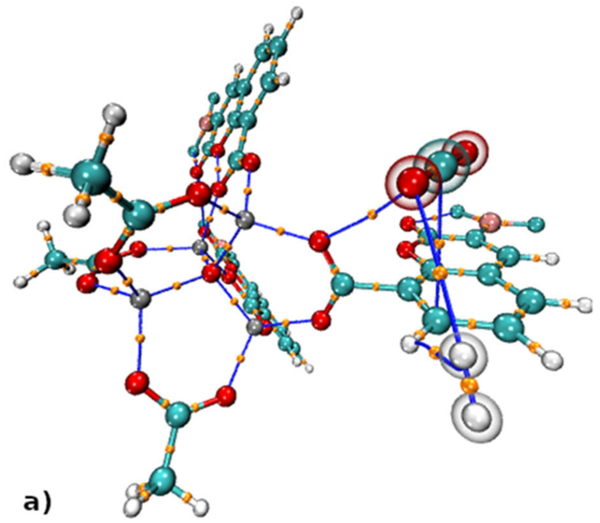

Figure SI-13. a) Electron density topologies for IRMOF-C-BF2---H2 CO2. Bond critical points (BCP) are shown in orange, paths are shown in blue. b) Laplacian of electron density isosurface in green (isovalue 0.3 a.u.**)**.

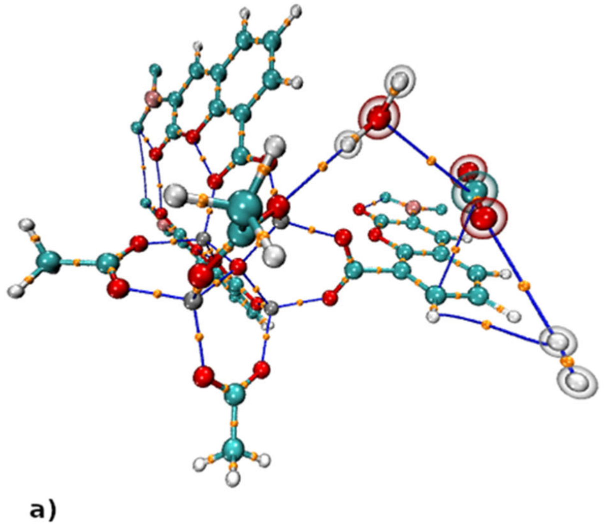

Figure SI-14. a) Electron density topologies for IRMOF-C-BF2---H2 CO2 H2O. Bond critical points (BCP) are shown in orange, paths are shown in blue. b) Laplacian of electron density isosurface in green (isovalue 0.3 a.u.).

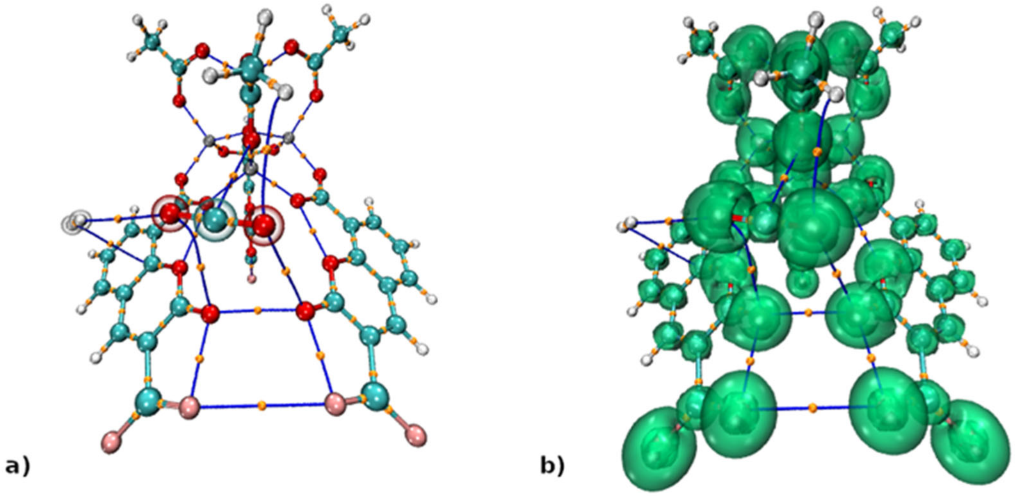

Figure SI-15. a) Electron density topologies for IRMOF-C-BF_2---CO_2 H_2. Bond critical points (BCP) are shown in orange, paths are shown in blue. b) Laplacian of electron density isosurface in green (isovalue 0.3 a.u.).

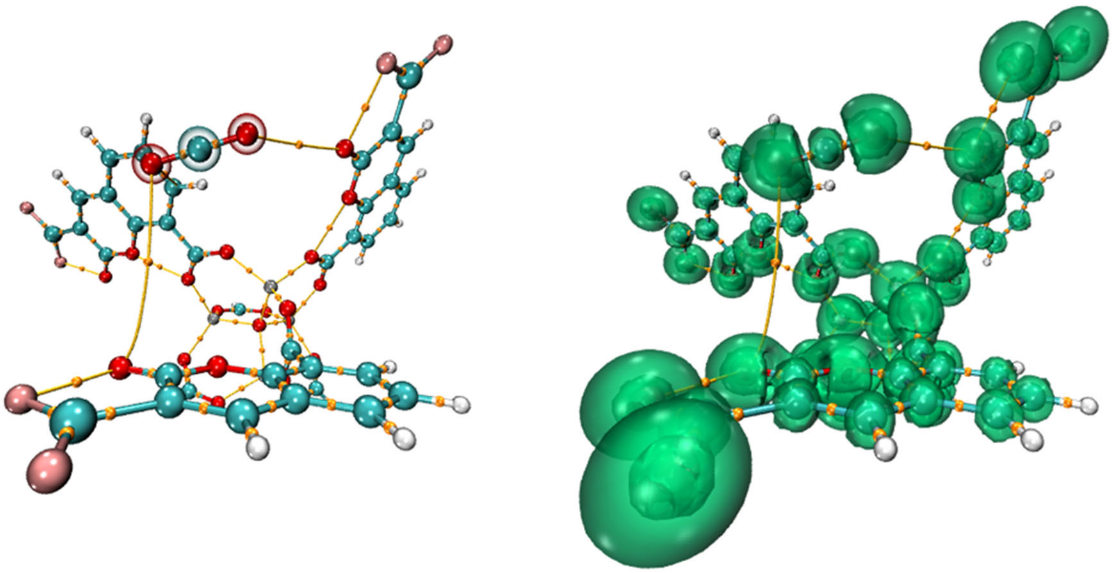

Figure SI-16. a) Electron density topologies for IRMOF-C-BF_2 unit cell CO_2. Bond critical points (BCP) are shown in orange, paths are shown in blue. b) Laplacian of electron density isosurface in green (isovalue 0.3 a.u.).

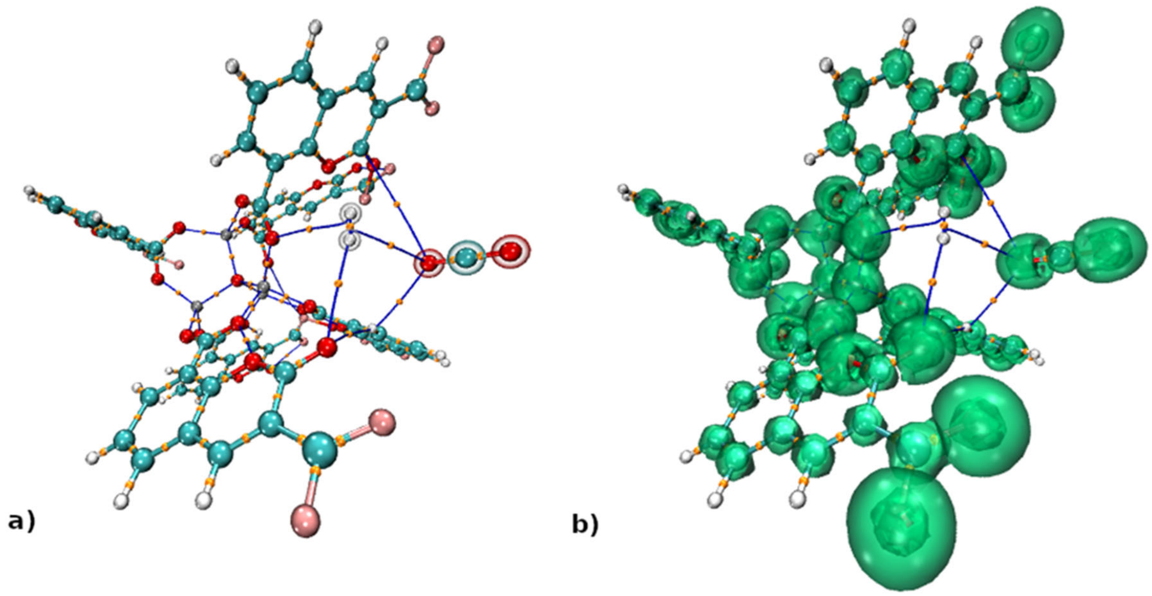

Figure SI-17. a) Electron density topologies for IRMOF-C-BF_2 unit cell CO_2 and H_2. Bond critical points (BCP) are shown in orange, paths are shown in blue. b) Laplacian of electron density isosurface in green (isovalue 0.3 a.u.).

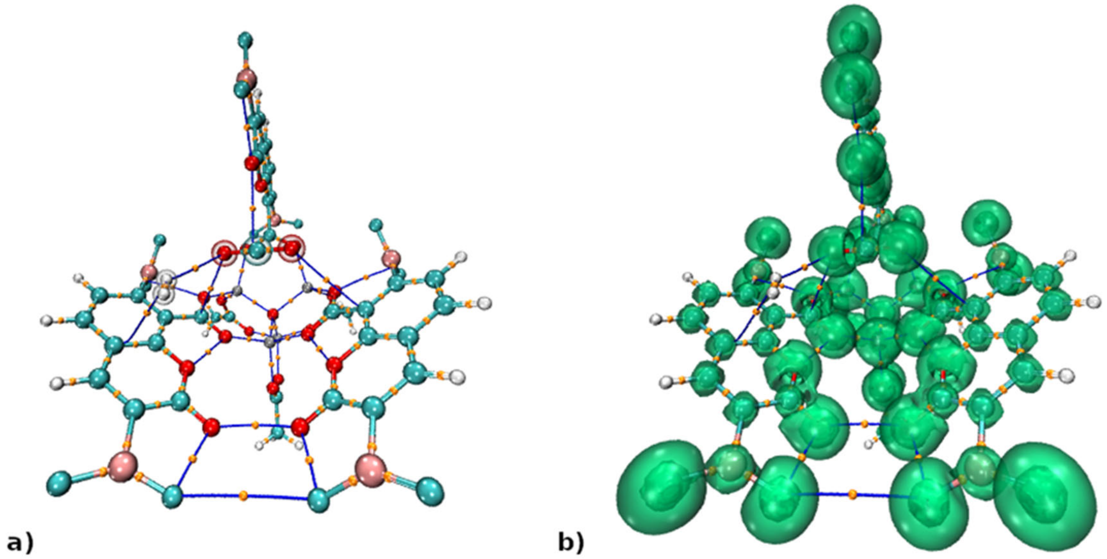

Figure SI-18. a) Electron density topologies for IRMOF-C-(2)-BF_2 unit cell CO_2 and H_2. Bond critical points (BCP) are shown in orange, paths are shown in blue. b) Laplacian of electron density isosurface in green (isovalue 0.3 a.u.).

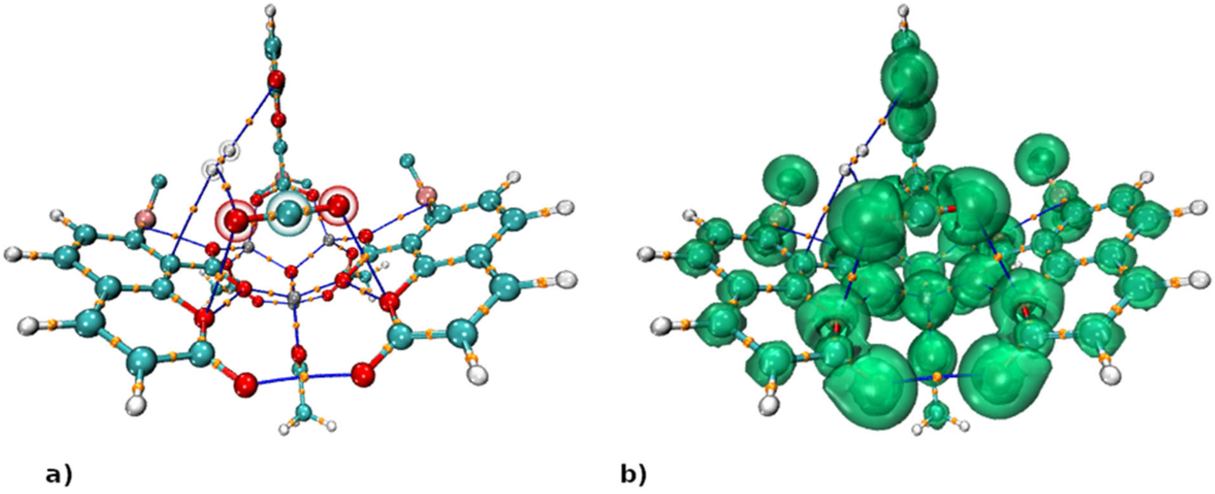

Figure SI-19. a) Electron density topologies for IRMOF-C′-BF_2---H_2 CO_2. Bond critical points (BCP) are shown in orange, paths are shown in blue. b) Laplacian of electron density isosurface in green (isovalue 0.3 a.u.).

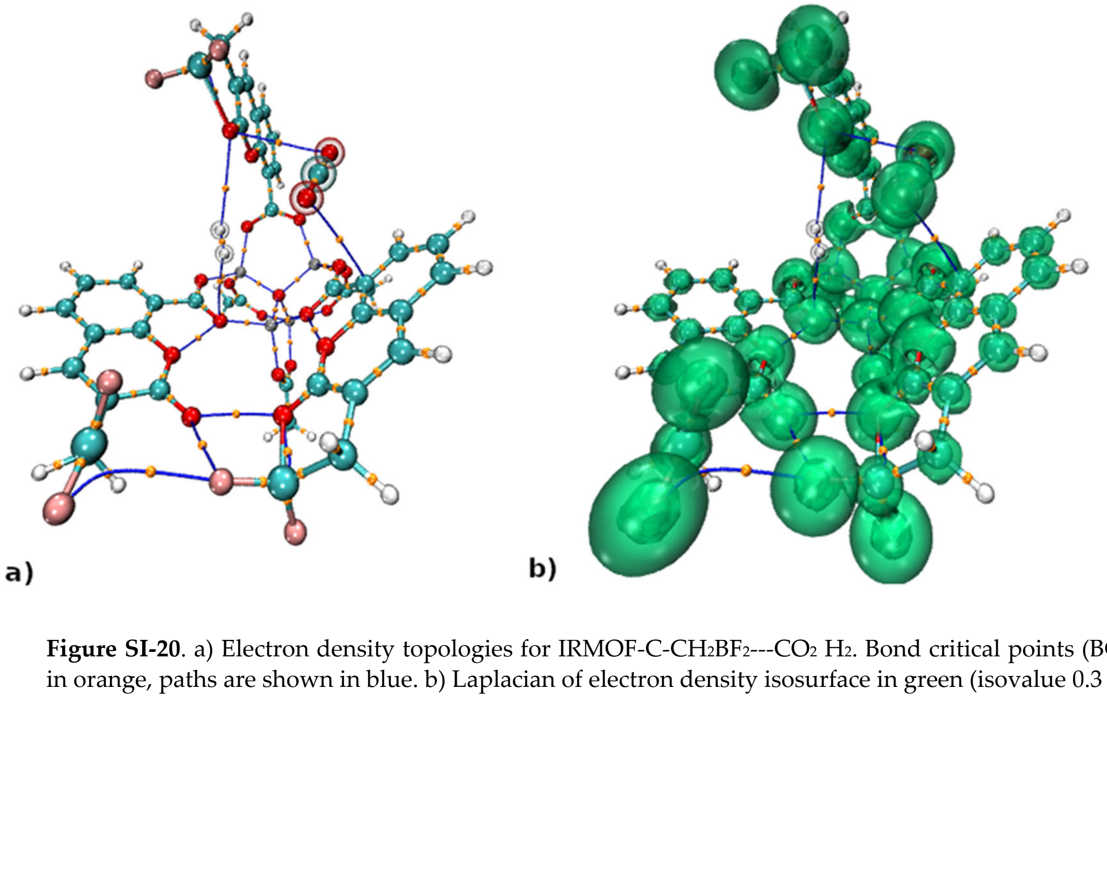

Figure SI-20. a) Electron density topologies for IRMOF-C-CH_2BF_2---CO_2 H_2. Bond critical points (BCP) are shown in orange, paths are shown in blue. b) Laplacian of electron density isosurface in green (isovalue 0.3 a.u.).

Table SI-1. Analysis of Quantum Theory Atoms in Molecules for hydrogen bonds. Distances d(H—O) in angstroms (Å), angles in degrees. The electron density ρ (e Å^{-3}), its Laplacian $\nabla^2\rho$ (e Å^{-5}) and the curvature λ3 (e Å^{-5}), and kinetic energy density (G) in kJ/mol, are the observed values at the (3, -1) CPs of the H---O interactions.

Structure	Guest	Angle	d(H—O)	ρ	$\nabla^2\rho$	G	λ3
IRMOF-C-BF_2···H_2O	H_2O	144.07	2.32	1.17	4.26	23.6	6.6
IRMOF-C-BF2···CO_2H_2O	CO_2	116.54	2.99	0.37	1.35	7.2	1.8
IRMOF-C-BF2···CO_2H_2O	H_{H2O}-O_{CO2}	169.24	2.34	0.85	3.25	17.3	4.9
IRMOF-C-BF_2···$H_2CO_2H_2O$	H_2O	163.80	2.03	1.88	7.68	43.3	12.2
IRMOF-C-BF_2···$H_2CO_2H_2O$	H_{H2}-O_{CO2}	174.57	2.82	0.37	1.32	7.2	1.9
IRMOF-C-BF2···H_2CO_2	H_{H2}-O_{CO2}	164.35	2.9	0.32	1.16	6.3	1.7
IRMOF-C-BF2···CO_2H_2	H_{H2}-O_{CO2}	157.24	3.16	0.16	0.71	3.5	1.0
IRMOF-C-BF2···CO_2H_2	CO_2	120.72	2.97	0.38	1.35	7.2	1.8
IRMOF-C-BF_2 unit cell CO_2H_2	CO_2	114.96	2.93	0.30	1.23	6.4	1.6
IRMOF-C-BF2 unit cell CO_2H_2	H_2	170.50	2.93	0.28	1.04	5.6	1.5
IRMOF-C-BF2 unit cell CO_2H_2	H_2	91.58	3.43	0.14	0.64	2.9	0.8
IRMOF-C-BF2 unit cell CO_2H_2	H_{H2}-O_{CO2}	89.14	3.4	0.17	0.74	3.5	1.0
IRMOF-C'-BF2	H_2	172.13	2.71	0.52	1.68	9.4	2.6
IRMOF-C'-BF2	H_{H2}-O_{CO2}	112.87	3.12	0.27	1.03	5.2	1.4
IRMOF-C-(2)-BF_2	H_2	141.45	2.81	0.43	1.47	8.1	2.2
IRMOF-C-CH2BF2···H_2CO_2	H_2	162.43	3.13	0.22	0.86	4.3	1.2
IRMOF-C-CH2BF2···H_2CO_2	H_2	135.92	2.92	0.43	1.43	7.7	2.0
IRMOF-C-CH2BF2···H_2CO_2	H_2	133.92	3.47	0.13	0.55	2.5	0.6
IRMOF-C-CH2BF2···H_2CO_2	H_{H2}-O_{CO2}	90.48	3.32	0.20	0.86	4.1	1.1

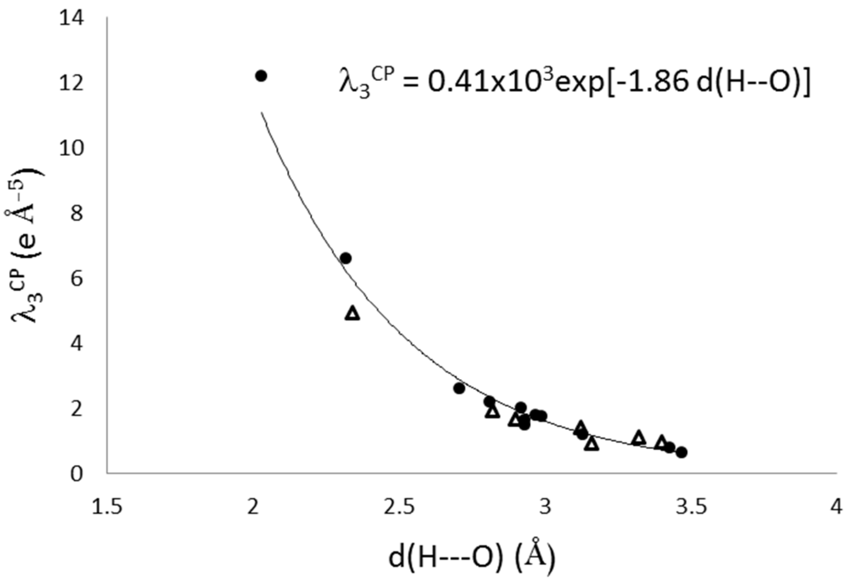

Figure SI-21. Behavior of λ_3^{CP} versus d(H---O). Dots are for hydrogen bonds between MOF´s and guest molecules. Triangles are for hydrogen bonds between guest molecules.

The xyz-coordinates of the geometries for the optimized molecules are listed below.

Figure1

74

Zn	1.12016501	-1.12325149	-1.12019609
Zn	1.12037274	1.11972698	1.12078807
Zn	-1.12081915	1.11972699	-1.12034163
Zn	-1.12229905	-1.12151003	1.12233020
O	0.00000000	0.00000000	0.00000000
O	3.01100604	-0.80403632	-0.80147751
O	3.01099823	0.80067974	0.80186059
O	0.80139398	-0.80403632	-3.01102828
O	-0.80194414	0.80067974	-3.01097599
O	-0.80157825	3.01062611	-0.80143281
O	0.80182977	0.80167246	3.01181160
O	-3.01343636	-0.80247816	0.80236640
O	-3.01183385	0.80167246	-0.80174622
O	-0.80228277	-0.80247816	3.01345863
O	0.80145506	3.01062611	0.80155601
O	-0.80250062	-3.01519368	0.80252290
O	0.80081161	-3.01458210	-0.80083384

O	1.70360822	1.70795132	5.08278285
O	3.28393460	3.28974847	5.11642038
O	1.70373736	5.07982997	-1.70378462
O	3.28476597	5.11421132	-3.28485710
O	5.08366535	-1.70207366	-1.70688627
O	5.11809784	-3.28320068	-3.28786852
B	3.63740627	3.64500347	7.80127747
B	7.80313857	-3.63559902	-3.64285075
B	3.63850564	7.79923490	-3.63860660
C	0.84306920	0.84777862	7.16872225
C	-0.86386200	-0.86072238	7.16559590
C	2.54272198	2.54819268	5.73887214
C	0.84605435	0.85000761	5.77821867
C	-0.86330397	-0.86091828	5.77515774
C	-0.00746748	-0.00469948	5.07589556
C	-0.00000543	-0.00012993	3.58358746
C	-0.00028013	-0.00165413	-3.58193008
C	0.03062197	5.19480361	-0.03062282
C	-0.86429207	5.77099820	0.86431603
C	-0.86479058	7.16144140	0.86481455
C	-0.01167845	7.85967882	0.01167877
C	0.84316968	7.16520783	-0.84319308
C	0.84582712	5.77486441	-0.84585059
C	-3.58358746	-0.00012992	0.00010487
C	2.54324950	5.73632012	-2.54332007
C	2.55563196	7.13583776	-2.55570286
C	1.70545382	7.85019549	-1.70550114
C	-0.00082649	-3.58526143	0.00082652
C	7.85400170	-1.70239656	-1.70988556
C	7.86344708	0.01486356	0.00715053
C	7.13967791	-2.55318744	-2.55989597
C	7.16916337	-0.84012436	-0.84706112
C	7.16516191	0.86767222	0.86056816
C	5.74017313	-2.54131957	-2.54666858
C	5.77865774	-0.84376092	-0.84933860
C	5.77472794	0.86645623	0.86070808
C	5.07589098	0.00986633	0.00489790
C	-0.00000000	3.58140162	-0.00000000
C	3.58193010	-0.00165412	0.00018075
C	2.55525160	2.56147944	7.13836914
C	1.70521779	1.71108139	7.85315513
C	-0.01114850	-0.00684860	7.86343812
F	9.21264276	-3.64778237	-3.65641860
F	4.37340043	6.84761056	-4.37352178
F	3.65140408	9.20873936	-3.65150538
F	3.65025117	3.65863745	9.21078313
F	4.37201919	4.37976460	6.84931897
F	6.85154831	-4.37102993	-4.37726874

H	1.71519826	1.72166619	8.94691269
H	-1.53255007	-1.53107667	5.22770105
H	-1.53384198	5.22322853	1.53388453
H	8.94779591	-1.71185866	-1.72044994
H	-1.52796471	-1.52510128	7.72586987
H	7.72509852	1.53236341	1.52463485
H	5.22692664	1.53578990	1.53050218
H	1.71547337	8.94397379	-1.71552096
H	-1.52915428	7.72140783	1.52919671

Figure2

89

Zn	1.11832588	-1.12823526	1.11702073
O	0.00000000	0.00000000	0.00000000
Zn	-1.11690035	1.12047199	1.12350596
Zn	1.12219395	1.11897262	-1.11971895
Zn	-1.12621595	-1.11650539	-1.12339591
O	0.80213697	-0.81178373	3.00875080
C	0.00305483	-0.00843257	3.58191925
O	-0.79708182	0.79703132	3.01323421
C	3.58191236	-0.01082909	-0.00354043
O	3.00968100	-0.81326198	0.79713438
O	3.01229190	0.79448044	-0.80316691
C	0.00918028	3.58138347	0.00676958
O	0.80842204	3.01007489	-0.79661456
O	-3.00902029	0.80786599	0.80608208
C	-0.00334651	0.00665239	-3.58357973
O	-0.80714607	-0.79471572	-3.01421696
O	0.80107402	0.80531462	-3.01104096
C	-3.58357456	0.00904983	0.00325300
O	-3.01623017	-0.79323645	-0.80105763
O	-0.79311094	3.01114733	0.80787288
C	-0.01001744	-3.58523958	-0.00760262
O	-0.81097469	-3.01160387	-0.80746843
O	0.79382786	-3.01813380	0.79438345
C	-0.00571474	-5.07589619	-0.00231149
H	8.94497845	-1.73802636	1.70882553
C	7.16655713	0.85094258	-0.86564050
B	7.79718631	-3.66246319	3.62865844
C	-4.98356264	0.00836246	-0.00248239
C	5.73601164	-2.56083027	2.53648029
C	5.77726518	-0.86016645	0.84232676
H	7.72757640	1.51545290	-1.52897402
H	5.22941714	1.52528967	-1.53249386
C	5.77612508	0.85328853	-0.86447976
F	6.84439910	-4.39684279	4.36257641

F	9.20666666	-3.67828270	3.64088242
C	-0.01166294	0.00503311	-4.98355409
C	5.07589281	-0.00312662	-0.00963575
H	8.95732133	-0.01270863	-0.00982188
C	7.16777284	-0.86008744	0.83875315
C	7.85120270	-1.72574250	1.69930405
C	7.86344925	-0.00526563	-0.01449110
C	0.04396636	5.19464087	0.04041330
C	-0.85030166	5.77481866	-0.85259583
C	-0.84723648	7.16525704	-0.85046567
C	0.00845759	7.85969098	0.00318860
C	0.86231933	7.16141583	0.85594479
C	0.86141534	5.77106764	0.85597177
O	1.71834058	5.07221542	1.71178622
C	2.56031505	5.72496288	2.55177400
C	2.57629640	7.12441826	2.56679053
C	1.72715998	7.84255923	1.71873767
O	3.30092537	5.09955437	3.29143861
B	3.66187609	7.78298868	3.64993108
F	4.39501394	6.82809579	4.38235721
F	3.67839951	9.19242854	3.66548201
H	-1.52187739	5.23003409	-1.52257081
H	-1.51078171	7.72817984	-1.51316532
H	0.01644550	8.95356000	0.01043066
H	1.73999253	8.93628735	1.73081555
C	1.71261932	-1.72163191	7.84921168
C	0.00000000	0.00000000	7.86346437
C	2.55979964	-2.57324399	7.13250833
C	0.85138892	-0.85585778	7.16678932
C	-0.85186005	0.85631198	7.16756877
C	2.54533688	-2.55869369	5.73304110
C	0.85239936	-0.85686860	5.77627780
C	-0.85326071	0.85772779	5.77713568
C	-0.00028150	0.00027256	5.07590291
O	1.70711511	-1.71605838	5.07888476
O	3.28406975	-3.30129242	5.10889086
H	1.72414872	-1.73319106	8.94297600
H	0.00668056	-0.00671498	8.95733072
B	3.64057594	-3.65967980	7.79293680
H	-1.51371401	1.52164117	7.72936440
H	-1.52183211	1.52981054	5.23120984
F	4.37224537	-4.39518736	6.83929068
F	3.65538739	-3.67456539	9.20240250
O	5.08087469	-1.71831757	1.69890139
O	5.11272795	-3.30251694	3.27685922
C	7.13549312	-2.57630803	2.54837377
H	0.87743529	-5.53817138	-0.41794072
H	-0.88618997	-5.54326193	-0.41792265

H	-0.00467563	-5.43227820	1.01719368
H	-5.33938817	0.00818571	-1.02218243
H	0.86749114	0.44073250	-5.43483467
H	-5.43045891	-0.87367249	0.43191152
H	-0.01380144	-1.01361636	-5.34236976
H	-5.43132480	0.88995991	0.43190797
H	-0.89611036	0.44074462	-5.42435816

Figure9_a

92

Zn	1.11832588	-1.12823526	1.11702073
O	0.00000000	0.00000000	0.00000000
Zn	-1.11690035	1.12047199	1.12350596
Zn	1.12219394	1.11897262	-1.11971895
Zn	-1.12621595	-1.11650539	-1.12339591
O	0.80213697	-0.81178373	3.00875080
C	0.00305483	-0.00843257	3.58191925
O	-0.79708182	0.79703132	3.01323421
C	3.58191236	-0.01082909	-0.00354043
O	3.00968100	-0.81326198	0.79713438
O	3.01229190	0.79448044	-0.80316691
C	0.00918028	3.58138347	0.00676958
O	0.80842204	3.01007489	-0.79661456
O	-3.00902029	0.80786599	0.80608208
C	-0.00334651	0.00665239	-3.58357973
O	-0.80714607	-0.79471572	-3.01421696
O	0.80107402	0.80531462	-3.01104096
C	-3.58357456	0.00904983	0.00325300
O	-3.01623016	-0.79323645	-0.80105763
O	-0.79311094	3.01114733	0.80787288
C	-0.01001744	-3.58523958	-0.00760262
O	-0.81097469	-3.01160387	-0.80746843
O	0.79382786	-3.01813380	0.79438345
C	-0.00571474	-5.07589619	-0.00231149
H	8.94497845	-1.73802636	1.70882553
C	7.16655713	0.85094258	-0.86564050
B	7.79718631	-3.66246319	3.62865844
C	-4.98356264	0.00836246	-0.00248239
C	5.73601164	-2.56083027	2.53648029
C	5.77726518	-0.86016645	0.84232676
H	7.72757640	1.51545290	-1.52897402
H	5.22941714	1.52528967	-1.53249386
C	5.77612508	0.85328853	-0.86447976
F	6.84439910	-4.39684279	4.36257641
F	9.20666666	-3.67828270	3.64088242
C	-0.01166294	0.00503311	-4.98355409

C	5.07589281	-0.00312662	-0.00963575
H	8.95732133	-0.01270863	-0.00982188
C	7.16777284	-0.86008744	0.83875315
C	7.85120270	-1.72574250	1.69930404
C	7.86344925	-0.00526563	-0.01449110
C	0.04396636	5.19464087	0.04041330
C	-0.85030166	5.77481866	-0.85259583
C	-0.84723648	7.16525704	-0.85046567
C	0.00845759	7.85969098	0.00318860
C	0.86231933	7.16141583	0.85594479
C	0.86141534	5.77106764	0.85597177
O	1.71834058	5.07221542	1.71178622
C	2.56031505	5.72496288	2.55177400
C	2.57629640	7.12441826	2.56679053
C	1.72715998	7.84255923	1.71873767
O	3.30092537	5.09955437	3.29143861
B	3.66187609	7.78298868	3.64993108
F	4.39501394	6.82809579	4.38235721
F	3.67839951	9.19242854	3.66548201
H	-1.52187739	5.23003409	-1.52257081
H	-1.51078171	7.72817984	-1.51316532
H	0.01644550	8.95356000	0.01043066
H	1.73999253	8.93628735	1.73081555
C	1.71261932	-1.72163191	7.84921168
C	0.00000000	0.00000000	7.86346437
C	2.55979964	-2.57324399	7.13250833
C	0.85138892	-0.85585778	7.16678932
C	-0.85186005	0.85631198	7.16756877
C	2.54533688	-2.55869369	5.73304110
C	0.85239936	-0.85686860	5.77627780
C	-0.85326071	0.85772779	5.77713568
C	-0.00028150	0.00027256	5.07590291
O	1.70711511	-1.71605838	5.07888476
O	3.28406975	-3.30129242	5.10889086
H	1.72414872	-1.73319106	8.94297600
H	0.00668056	-0.00671498	8.95733072
B	3.64057594	-3.65967980	7.79293679
H	-1.51371400	1.52164117	7.72936440
H	-1.52183211	1.52981054	5.23120984
F	4.37224537	-4.39518736	6.83929068
F	3.65538739	-3.67456539	9.20240250
O	5.08087469	-1.71831757	1.69890138
O	5.11272795	-3.30251694	3.27685922
C	7.13549312	-2.57630803	2.54837377
C	2.93841364	-4.97687723	1.86860666
O	3.63364160	-4.70078713	0.96587711
O	2.25068866	-5.27280136	2.76974291
H	-5.33938817	0.00818571	-1.02218243

H	0.86749114	0.44073250	-5.43483467
H	-5.43045891	-0.87367249	0.43191152
H	-0.01380144	-1.01361636	-5.34236976
H	-5.43132480	0.88995991	0.43190797
H	-0.89611036	0.44074462	-5.42435816
H	0.87743529	-5.53817138	-0.41794072
H	-0.88618997	-5.54326193	-0.41792265
H	-0.00467563	-5.43227820	1.01719368

Figure9_b

91

Zn	1.11832588	-1.12823526	1.11702073
O	0.00000000	0.00000000	0.00000000
Zn	-1.11690035	1.12047199	1.12350596
Zn	1.12219394	1.11897262	-1.11971895
Zn	-1.12621595	-1.11650539	-1.12339591
O	0.80213697	-0.81178373	3.00875080
C	0.00305483	-0.00843257	3.58191925
O	-0.79708182	0.79703132	3.01323421
C	3.58191236	-0.01082909	-0.00354043
O	3.00968100	-0.81326198	0.79713438
O	3.01229190	0.79448044	-0.80316691
C	0.00918028	3.58138347	0.00676958
O	0.80842204	3.01007489	-0.79661456
O	-3.00902029	0.80786599	0.80608208
C	-0.00334651	0.00665239	-3.58357973
O	-0.80714607	-0.79471572	-3.01421696
O	0.80107402	0.80531462	-3.01104096
C	-3.58357456	0.00904983	0.00325300
O	-3.01623016	-0.79323645	-0.80105763
O	-0.79311094	3.01114733	0.80787288
C	-0.01001744	-3.58523958	-0.00760262
O	-0.81097469	-3.01160387	-0.80746843
O	0.79382786	-3.01813380	0.79438345
C	-0.00571474	-5.07589619	-0.00231149
H	8.94497845	-1.73802636	1.70882553
C	7.16655713	0.85094258	-0.86564050
B	7.79718631	-3.66246319	3.62865844
C	-4.98356264	0.00836246	-0.00248239
C	5.73601164	-2.56083027	2.53648029
C	5.77726518	-0.86016645	0.84232676
H	7.72757640	1.51545290	-1.52897402
H	5.22941714	1.52528967	-1.53249386
C	5.77612508	0.85328853	-0.86447976
F	6.84439910	-4.39684279	4.36257641
F	9.20666666	-3.67828270	3.64088242

C	-0.01166294	0.00503311	-4.98355409
C	5.07589281	-0.00312662	-0.00963575
H	8.95732133	-0.01270863	-0.00982188
C	7.16777284	-0.86008744	0.83875315
C	7.85120270	-1.72574250	1.69930404
C	7.86344925	-0.00526563	-0.01449110
C	0.04396636	5.19464087	0.04041330
C	-0.85030166	5.77481866	-0.85259583
C	-0.84723648	7.16525704	-0.85046567
C	0.00845759	7.85969098	0.00318860
C	0.86231933	7.16141583	0.85594479
C	0.86141534	5.77106764	0.85597177
O	1.71834058	5.07221542	1.71178622
C	2.56031505	5.72496288	2.55177400
C	2.57629640	7.12441826	2.56679053
C	1.72715998	7.84255923	1.71873767
O	3.30092537	5.09955437	3.29143861
B	3.66187609	7.78298868	3.64993108
F	4.39501394	6.82809579	4.38235721
F	3.67839951	9.19242854	3.66548201
H	-1.52187739	5.23003409	-1.52257081
H	-1.51078171	7.72817984	-1.51316532
H	0.01644550	8.95356000	0.01043066
H	1.73999253	8.93628735	1.73081555
C	1.71261932	-1.72163191	7.84921168
C	0.00000000	0.00000000	7.86346437
C	2.55979964	-2.57324399	7.13250833
C	0.85138892	-0.85585778	7.16678932
C	-0.85186005	0.85631198	7.16756877
C	2.54533688	-2.55869369	5.73304110
C	0.85239936	-0.85686860	5.77627780
C	-0.85326071	0.85772779	5.77713568
C	-0.00028150	0.00027256	5.07590291
O	1.70711511	-1.71605838	5.07888476
O	3.28406975	-3.30129242	5.10889086
H	1.72414872	-1.73319106	8.94297600
H	0.00668056	-0.00671498	8.95733072
B	3.64057594	-3.65967980	7.79293679
H	-1.51371400	1.52164117	7.72936440
H	-1.52183211	1.52981054	5.23120984
F	4.37224537	-4.39518736	6.83929068
F	3.65538739	-3.67456539	9.20240250
O	5.08087469	-1.71831757	1.69890138
O	5.11272795	-3.30251694	3.27685922
C	7.13549312	-2.57630803	2.54837377
H	0.87743529	-5.53817138	-0.41794072
H	-0.88618997	-5.54326193	-0.41792265
H	-0.00467563	-5.43227820	1.01719368

H	-5.33938817	0.00818571	-1.02218243
H	0.86749114	0.44073250	-5.43483467
H	-5.43045891	-0.87367249	0.43191152
H	-0.01380144	-1.01361636	-5.34236976
H	-5.43132480	0.88995991	0.43190797
H	-0.89611036	0.44074462	-5.42435816
H	0.93193216	6.46079504	-3.20449546
H	1.09538841	6.39684480	-3.93783079

Figure9_c

92

Zn	1.11832588	-1.12823526	1.11702073
O	0.00000000	0.00000000	0.00000000
Zn	-1.11690035	1.12047199	1.12350596
Zn	1.12219395	1.11897262	-1.11971895
Zn	-1.12621595	-1.11650539	-1.12339591
O	0.80213697	-0.81178373	3.00875080
C	0.00305483	-0.00843257	3.58191925
O	-0.79708182	0.79703132	3.01323421
C	3.58191236	-0.01082909	-0.00354043
O	3.00968100	-0.81326198	0.79713438
O	3.01229190	0.79448044	-0.80316691
C	0.00918028	3.58138347	0.00676958
O	0.80842204	3.01007489	-0.79661456
O	-3.00902029	0.80786599	0.80608208
C	-0.00334651	0.00665239	-3.58357973
O	-0.80714607	-0.79471572	-3.01421696
O	0.80107402	0.80531462	-3.01104096
C	-3.58357456	0.00904983	0.00325300
O	-3.01623017	-0.79323645	-0.80105763
O	-0.79311094	3.01114733	0.80787288
C	-0.01001744	-3.58523958	-0.00760262
O	-0.81097469	-3.01160387	-0.80746843
O	0.79382786	-3.01813380	0.79438345
C	-0.00571474	-5.07589619	-0.00231149
H	8.94497845	-1.73802636	1.70882553
C	7.16655713	0.85094258	-0.86564050
B	7.79718631	-3.66246319	3.62865844
C	-4.98356264	0.00836246	-0.00248239
C	5.73601164	-2.56083027	2.53648029
C	5.77726518	-0.86016645	0.84232676
H	7.72757640	1.51545290	-1.52897402
H	5.22941714	1.52528967	-1.53249386
C	5.77612508	0.85328853	-0.86447976
F	6.84439910	-4.39684279	4.36257641
F	9.20666666	-3.67828270	3.64088242

C	-0.01166294	0.00503311	-4.98355409
C	5.07589281	-0.00312662	-0.00963575
H	8.95732133	-0.01270863	-0.00982188
C	7.16777284	-0.86008744	0.83875315
C	7.85120270	-1.72574250	1.69930405
C	7.86344925	-0.00526563	-0.01449110
C	0.04396636	5.19464087	0.04041330
C	-0.85030166	5.77481866	-0.85259583
C	-0.84723648	7.16525704	-0.85046567
C	0.00845759	7.85969098	0.00318860
C	0.86231933	7.16141583	0.85594479
C	0.86141534	5.77106764	0.85597177
O	1.71834058	5.07221542	1.71178622
C	2.56031505	5.72496288	2.55177400
C	2.57629640	7.12441826	2.56679053
C	1.72715998	7.84255923	1.71873767
O	3.30092537	5.09955437	3.29143861
B	3.66187609	7.78298868	3.64993108
F	4.39501394	6.82809579	4.38235721
F	3.67839951	9.19242854	3.66548201
H	-1.52187739	5.23003409	-1.52257081
H	-1.51078171	7.72817984	-1.51316532
H	0.01644550	8.95356000	0.01043066
H	1.73999253	8.93628735	1.73081555
C	1.71261932	-1.72163191	7.84921168
C	0.00000000	0.00000000	7.86346437
C	2.55979964	-2.57324399	7.13250833
C	0.85138892	-0.85585778	7.16678932
C	-0.85186005	0.85631198	7.16756877
C	2.54533688	-2.55869369	5.73304110
C	0.85239936	-0.85686860	5.77627780
C	-0.85326071	0.85772779	5.77713568
C	-0.00028150	0.00027256	5.07590291
O	1.70711511	-1.71605838	5.07888476
O	3.28406975	-3.30129242	5.10889086
H	1.72414872	-1.73319106	8.94297600
H	0.00668056	-0.00671498	8.95733072
B	3.64057594	-3.65967980	7.79293680
H	-1.51371401	1.52164117	7.72936440
H	-1.52183211	1.52981054	5.23120984
F	4.37224537	-4.39518736	6.83929068
F	3.65538739	-3.67456539	9.20240250
O	5.08087469	-1.71831757	1.69890139
O	5.11272795	-3.30251694	3.27685922
C	7.13549312	-2.57630803	2.54837377
H	0.87743529	-5.53817138	-0.41794072
H	-0.88618997	-5.54326193	-0.41792265
H	-0.00467563	-5.43227820	1.01719368

H	-5.33938817	0.00818571	-1.02218243
H	0.86749114	0.44073250	-5.43483467
H	-5.43045891	-0.87367249	0.43191152
H	-0.01380144	-1.01361636	-5.34236976
H	-5.43132480	0.88995991	0.43190797
H	-0.89611036	0.44074462	-5.42435816
O	-1.22600885	10.84609192	-0.50368107
H	-2.17657874	10.84917299	-0.27654329
H	-1.12659059	11.62068222	-1.09073389

Figure9_d

94

Zn	1.11832588	-1.12823526	1.11702073
O	0.00000000	0.00000000	0.00000000
Zn	-1.11690035	1.12047199	1.12350596
Zn	1.12219395	1.11897262	-1.11971895
Zn	-1.12621595	-1.11650539	-1.12339591
O	0.80213697	-0.81178373	3.00875080
C	0.00305483	-0.00843257	3.58191925
O	-0.79708182	0.79703132	3.01323421
C	3.58191236	-0.01082909	-0.00354043
O	3.00968100	-0.81326198	0.79713438
O	3.01229190	0.79448044	-0.80316691
C	0.00918028	3.58138347	0.00676958
O	0.80842204	3.01007489	-0.79661456
O	-3.00902029	0.80786599	0.80608208
C	-0.00334651	0.00665239	-3.58357973
O	-0.80714607	-0.79471572	-3.01421696
O	0.80107402	0.80531462	-3.01104096
C	-3.58357456	0.00904983	0.00325300
O	-3.01623017	-0.79323645	-0.80105763
O	-0.79311094	3.01114733	0.80787288
C	-0.01001744	-3.58523958	-0.00760262
O	-0.81097469	-3.01160387	-0.80746843
O	0.79382786	-3.01813380	0.79438345
C	-0.00571474	-5.07589619	-0.00231149
H	8.94497845	-1.73802636	1.70882553
C	7.16655713	0.85094258	-0.86564050
B	7.79718631	-3.66246319	3.62865844
C	-4.98356264	0.00836246	-0.00248239
C	5.73601164	-2.56083027	2.53648029
C	5.77726518	-0.86016645	0.84232676
H	7.72757640	1.51545290	-1.52897402
H	5.22941714	1.52528967	-1.53249386
C	5.77612508	0.85328853	-0.86447976
F	6.84439910	-4.39684279	4.36257641

F	9.20666666	-3.67828270	3.64088242
C	-0.01166294	0.00503311	-4.98355409
C	5.07589281	-0.00312662	-0.00963575
H	8.95732133	-0.01270863	-0.00982188
C	7.16777284	-0.86008744	0.83875315
C	7.85120270	-1.72574250	1.69930405
C	7.86344925	-0.00526563	-0.01449110
C	0.04396636	5.19464087	0.04041330
C	-0.85030166	5.77481866	-0.85259583
C	-0.84723648	7.16525704	-0.85046567
C	0.00845759	7.85969098	0.00318860
C	0.86231933	7.16141583	0.85594479
C	0.86141534	5.77106764	0.85597177
O	1.71834058	5.07221542	1.71178622
C	2.56031505	5.72496288	2.55177400
C	2.57629640	7.12441826	2.56679053
C	1.72715998	7.84255923	1.71873767
O	3.30092537	5.09955437	3.29143861
B	3.66187609	7.78298868	3.64993108
F	4.39501394	6.82809579	4.38235721
F	3.67839951	9.19242854	3.66548201
H	-1.52187739	5.23003409	-1.52257081
H	-1.51078171	7.72817984	-1.51316532
H	0.01644550	8.95356000	0.01043066
H	1.73999253	8.93628735	1.73081555
C	1.71261932	-1.72163191	7.84921168
C	0.00000000	0.00000000	7.86346437
C	2.55979964	-2.57324399	7.13250833
C	0.85138892	-0.85585778	7.16678932
C	-0.85186005	0.85631198	7.16756877
C	2.54533688	-2.55869369	5.73304110
C	0.85239936	-0.85686860	5.77627780
C	-0.85326071	0.85772779	5.77713568
C	-0.00028150	0.00027256	5.07590291
O	1.70711511	-1.71605838	5.07888476
O	3.28406975	-3.30129242	5.10889086
H	1.72414872	-1.73319106	8.94297600
H	0.00668056	-0.00671498	8.95733072
B	3.64057594	-3.65967980	7.79293680
H	-1.51371401	1.52164117	7.72936440
H	-1.52183211	1.52981054	5.23120984
F	4.37224537	-4.39518736	6.83929068
F	3.65538739	-3.67456539	9.20240250
O	5.08087469	-1.71831757	1.69890139
O	5.11272795	-3.30251694	3.27685922
C	7.13549312	-2.57630803	2.54837377
H	0.87743529	-5.53817138	-0.41794072
H	-0.88618997	-5.54326193	-0.41792265

H	-0.00467563	-5.43227820	1.01719368
H	-5.33938817	0.00818571	-1.02218243
H	0.86749114	0.44073250	-5.43483467
H	-5.43045891	-0.87367249	0.43191152
H	-0.01380144	-1.01361636	-5.34236976
H	-5.43132480	0.88995991	0.43190797
H	-0.89611036	0.44074462	-5.42435816
H	-2.72672531	6.01382727	-4.00272313
H	-2.00313474	5.83105120	-4.11483109
C	1.55557907	5.57918534	-3.04281723
O	0.77370776	5.07748562	-3.75783044
O	2.33734353	6.09060182	-2.33586498

Figure9_e

95

Zn	-0.15577398	-1.67446409	-0.13151325
O	-1.00849389	-1.15558820	-1.79732819
Zn	-2.62703734	-0.15669243	-1.41299255
Zn	0.20610823	-0.04927571	-2.82980281
Zn	-1.45781369	-2.74814787	-2.81655850
O	-1.15364313	-1.05038092	1.41559277
C	-2.23282131	-0.38424002	1.47929665
O	-2.92167486	0.03544666	0.49861461
C	2.29553838	-0.21255044	-0.78526275
O	1.63958161	-0.94447777	0.01874628
O	1.89835898	0.21819486	-1.91180632
C	-1.65407446	2.21146601	-2.83299672
O	-0.53800584	1.71323736	-3.17470713
O	-4.20491710	-1.02316513	-2.14655839
C	0.21645825	-1.92904662	-5.07503370
O	-0.57374469	-2.77191869	-4.54815181
O	0.61602851	-0.84038237	-4.55743527
C	-4.31363558	-2.10080195	-2.80960709
O	-3.36891810	-2.87789573	-3.15033083
O	-2.56443952	1.63640655	-2.16132044
C	-0.36269733	-4.52666754	-0.76153291
O	-0.93082033	-4.37514242	-1.88610045
O	-0.00022540	-3.60573670	0.03311064
C	-0.08928661	-5.92418936	-0.32075746
H	6.96470163	-0.03399441	2.79871797
C	5.73780292	1.36405098	-0.81149069
B	5.59862674	-1.72961704	4.79003554
C	-5.60167708	-2.47555492	-3.21024317
C	3.87336077	-1.48143377	2.88825962
C	4.18303269	-0.24160728	0.85576139
H	6.36141461	1.99332263	-1.45293716

H	4.05869801	1.33645630	-2.16492854
C	4.45565009	0.99619027	-1.20400917
F	4.60249068	-2.51350037	5.40565905
F	6.89619378	-1.36642678	5.20409733
C	0.68931104	-2.23703653	-6.35626453
C	3.67297850	0.19125213	-0.37092442
H	7.25164701	1.21596057	0.72847705
C	5.46564158	0.12802637	1.24538906
C	5.95778291	-0.31583543	2.47736574
C	6.24385599	0.93060257	0.41294355
C	-1.92712928	3.74298970	-3.26290101
C	-2.55048143	3.85556393	-4.50086143
C	-2.80141098	5.16254985	-4.90354613
C	-2.43205247	6.22808755	-4.08416822
C	-1.81063772	5.98510311	-2.85996912
C	-1.55847293	4.67924627	-2.45472876
O	-0.93517910	4.43720047	-1.22670095
C	-0.56618989	5.45696794	-0.41153164
C	-0.81127793	6.77866195	-0.80142018
C	-1.43356656	7.04258229	-2.02577948
O	-0.02360688	5.22766927	0.65606800
B	-0.30226506	7.92162134	0.30308942
F	0.29587234	7.37935340	1.45805120
F	-0.54885380	9.25295188	-0.08906939
H	-2.84040729	3.01951140	-5.14400204
H	-3.28800592	5.37042218	-5.86081288
H	-2.62623117	7.25899877	-4.39430109
H	-1.62491392	8.07570243	-2.33008313
C	-1.80942027	-0.61032348	6.36161836
C	-3.70583504	0.55336703	5.38862302
C	-0.62790022	-1.34016851	6.19441208
C	-2.52653417	-0.17499136	5.24214682
C	-4.40797037	0.97938111	4.26224635
C	-0.16382786	-1.63450093	4.90719233
C	-2.04844305	-0.47786930	3.97202156
C	-3.93256965	0.67815681	2.99080378
C	-2.74994918	-0.05226866	2.84107778
O	-0.86522378	-1.20862665	3.82680102
O	0.86616904	-2.27074431	4.76212890
H	-2.17187730	-0.38040139	7.36777324
H	-4.07367694	0.78656887	6.39208828
B	0.33923133	-1.92902332	7.42017408
H	-5.33167085	1.54998751	4.39458566
H	-4.48371662	1.01257666	2.10699477
F	1.47445297	-2.63209773	6.96994009
F	-0.12788837	-1.63273992	8.71672715
O	3.40362369	-1.04677630	1.69201949
O	3.17998623	-2.18301755	3.60502637

C	5.16176471	-1.12065743	3.29909434
C	2.20079033	-4.40789587	1.94092671
O	2.95941828	-4.05693760	1.11245712
O	1.45671199	-4.81084615	2.74608517
H	-5.57977003	-2.79209840	-4.24258048
H	1.57499298	-1.69181783	-6.64730419
H	-6.00200564	-3.32845605	-2.68232584
H	0.99578964	-3.27216109	-6.38769745
H	-6.32373746	-1.67252881	-3.19690683
H	-0.05381829	-2.15923238	-7.13607560
H	0.95125649	-6.21341137	-0.31620949
H	-0.67306066	-6.68789687	-0.81305685
H	-0.37318949	-6.03700952	0.71513383
O	2.44294726	-3.03512121	-1.99294949
H	2.54936475	-3.22178678	-1.03845862
H	3.13483287	-3.57865771	-2.41333965

Figure9_f

97

Zn	0.93922116	-1.09691100	1.31325872
O	-0.16775222	0.03614049	0.18982331
Zn	-1.10499581	1.32668215	1.29493244
Zn	0.95149763	0.97076554	-1.09038968
Zn	-1.45992531	-1.06120932	-0.76048040
O	0.79248013	-0.61619027	3.19105911
C	0.10467870	0.29180728	3.75221602
O	-0.67009450	1.11773771	3.17779561
C	3.39167605	-0.27707223	-0.06036481
O	2.81999626	-0.96709180	0.83934772
O	2.82862017	0.51221636	-0.88035567
C	0.12408151	3.59468267	-0.08954783
O	0.81296846	2.90026491	-0.89814272
O	-3.03439463	1.15225376	1.13495365
C	-0.44073855	-0.22139317	-3.37405856
O	-1.25996125	-0.90788639	-2.68844951
O	0.46498186	0.54661786	-2.92396527
C	-3.72899466	0.34770419	0.43998230
O	-3.28889186	-0.55674006	-0.33509747
O	-0.65795757	3.15483757	0.80798108
C	-0.46078477	-3.52622435	0.46873127
O	-1.27207063	-2.94765057	-0.31704439
O	0.44341735	-2.97133166	1.16548841
C	-0.57356622	-5.00746091	0.59251430
H	8.71576281	-2.31949271	1.40950058
C	6.95784658	0.21323531	-1.23406466
B	7.56792486	-3.99330803	3.55133453

C	-5.12111926	0.46470896	0.53108087
C	5.52350305	-2.80531404	2.51987976
C	5.57078494	-1.24385530	0.69677074
H	7.51786829	0.77728146	-1.98543379
H	5.03511870	0.99755555	-1.81708099
C	5.57597995	0.33295693	-1.13701059
F	6.61828818	-4.58852312	4.40553685
F	8.96867367	-4.12703771	3.46734810
C	-0.55468402	-0.32560244	-4.76551710
C	4.87689320	-0.39590881	-0.17028267
H	8.73440238	-0.73290369	-0.43793289
C	6.95273588	-1.36135166	0.59711874
C	7.62876080	-2.21571165	1.47463925
C	7.64728901	-0.63356682	-0.36757651
C	0.28831451	5.19732270	-0.18708691
C	-0.62223263	5.78339669	-1.05955930
C	-0.50946449	7.16497249	-1.16848332
C	0.46020561	7.84582930	-0.43407854
C	1.31824702	7.14284081	0.41057125
C	1.20779692	5.76133145	0.52148383
O	2.06906867	5.05773673	1.36900910
C	3.02078589	5.69731876	2.09409454
C	3.14807524	7.08771594	1.99637366
C	2.29665090	7.81039349	1.15449093
O	3.76346641	5.06794746	2.82830201
B	4.36073605	7.73047416	2.94595639
F	5.06948094	6.77379087	3.69974503
F	4.48939125	9.13078660	2.84793362
H	-1.38324230	5.24926641	-1.63596178
H	-1.17466039	7.73142928	-1.82650027
H	0.55488412	8.93266633	-0.51462012
H	2.39649866	8.89703860	1.07843462
C	1.99078634	-1.23994933	8.01411314
C	0.42511360	0.61637262	8.00940851
C	2.71177451	-2.21055276	7.31074525
C	1.15146074	-0.35732694	7.32600474
C	-0.40665893	1.48780777	7.30800872
C	2.59303170	-2.29813836	5.91892689
C	1.04754791	-0.46102021	5.94326319
C	-0.51277137	1.38673505	5.92531837
C	0.21468709	0.41097763	5.23739973
O	1.77688537	-1.43836443	5.25918112
O	3.22178705	-3.14443673	5.30640064
H	2.08380052	-1.17170126	9.10189873
H	0.51369724	0.68985095	9.09724452
B	3.75029112	-3.33259147	7.97939933
H	-0.96978366	2.24623649	7.85937461
H	-1.16555796	2.07070455	5.37506992

F	4.34774159	-4.19556040	7.03913873
F	3.87010796	-3.24463068	9.38116299
O	4.87551075	-1.97464248	1.66509250
O	4.90131596	-3.43511222	3.35831885
C	6.91431463	-2.93788495	2.43622842
H	0.23655469	-5.57199950	0.15502112
H	-1.51695141	-5.42826711	0.27732144
H	-0.52374912	-5.28645741	1.63466555
H	-5.55171671	0.41931080	-0.45832543
H	0.31953341	-0.00011811	-5.30976321
H	-5.60209956	-0.34201235	1.06424003
H	-0.66412793	-1.36412940	-5.04098720
H	-5.46399380	1.41058019	0.92371895
H	-1.43275847	0.14945449	-5.17747119
H	-2.90456072	6.08637323	-4.67530778
H	-2.18730610	5.86028500	-4.60810615
C	1.38969471	5.21421363	-3.57309585
O	0.41369671	4.88533991	-4.13726248
O	2.34719628	5.58466906	-3.01050022
O	2.47979783	2.45778151	-4.03307573
H	2.58432722	2.41627232	-5.00224983
H	1.76366994	1.81015179	-3.84689259

Figure10_a

77

Zn	1.12016501	-1.12325149	-1.12019609
Zn	1.12037274	1.11972698	1.12078807
Zn	-1.12081915	1.11972699	-1.12034163
Zn	-1.12229905	-1.12151003	1.12233020
O	0.00000000	0.00000000	0.00000000
O	3.01100604	-0.80403632	-0.80147751
O	3.01099823	0.80067974	0.80186059
O	0.80139398	-0.80403632	-3.01102828
O	-0.80194414	0.80067974	-3.01097599
O	-0.80157825	3.01062611	-0.80143281
O	0.80182977	0.80167246	3.01181160
O	-3.01343636	-0.80247816	0.80236640
O	-3.01183385	0.80167246	-0.80174622
O	-0.80228277	-0.80247816	3.01345863
O	0.80145506	3.01062611	0.80155601
O	-0.80250062	-3.01519368	0.80252290
O	0.80081161	-3.01458210	-0.80083384
O	1.70360822	1.70795132	5.08278285
O	3.28393460	3.28974847	5.11642038

O	1.70373736	5.07982997	-1.70378462
O	3.28476597	5.11421132	-3.28485710
O	5.08366535	-1.70207366	-1.70688627
O	5.11809784	-3.28320068	-3.28786852
O	6.25482341	4.42804722	0.40041421
O	5.32295707	4.88179086	2.50724949
C	2.55525160	2.56147944	7.13836914
C	0.84306920	0.84777862	7.16872225
C	-0.86386200	-0.86072238	7.16559590
C	2.54272198	2.54819268	5.73887214
C	0.84605435	0.85000761	5.77821867
C	-0.86330397	-0.86091828	5.77515774
C	-0.00746748	-0.00469948	5.07589556
C	-0.00000543	-0.00012993	3.58358746
C	-0.00028013	-0.00165413	-3.58193008
C	0.03062197	5.19480361	-0.03062282
C	-0.86429207	5.77099820	0.86431603
C	-0.86479058	7.16144140	0.86481455
C	-0.01167845	7.85967882	0.01167877
C	0.84316968	7.16520783	-0.84319308
C	0.84582712	5.77486441	-0.84585059
C	-3.58358746	-0.00012992	0.00010487
C	2.54324950	5.73632012	-2.54332007
C	2.55563196	7.13583776	-2.55570286
C	1.70545382	7.85019549	-1.70550114
C	-0.00082649	-3.58526143	0.00082652
C	7.85400170	-1.70239656	-1.70988556
C	7.86344708	0.01486356	0.00715053
C	7.13967791	-2.55318744	-2.55989597
C	7.16916337	-0.84012436	-0.84706112
C	7.16516191	0.86767222	0.86056816
C	5.74017313	-2.54131957	-2.54666858
C	5.77865774	-0.84376092	-0.84933860
C	5.77472794	0.86645623	0.86070808
C	5.07589098	0.00986633	0.00489790
C	-0.00000000	3.58140162	-0.00000000
C	3.58193010	-0.00165412	0.00018075
C	5.78834970	4.65495211	1.45435794
C	1.70521779	1.71108139	7.85315513
C	-0.01114850	-0.00684860	7.86343812
F	9.21264276	-3.64778237	-3.65641860
F	4.37340043	6.84761056	-4.37352178
F	3.65140408	9.20873936	-3.65150538
F	3.65025117	3.65863745	9.21078313
F	4.37201919	4.37976460	6.84931897
F	6.85154831	-4.37102993	-4.37726874
H	1.71519826	1.72166619	8.94691269
H	-1.53255007	-1.53107667	5.22770105

H	-1.53384198	5.22322853	1.53388453
H	8.94779591	-1.71185866	-1.72044994
H	-1.52796471	-1.52510128	7.72586987
H	7.72509852	1.53236341	1.52463485
H	5.22692664	1.53578990	1.53050218
H	1.71547337	8.94397379	-1.71552096
H	-1.52915428	7.72140783	1.52919671
B	3.63740627	3.64500347	7.80127747
B	7.80313857	-3.63559902	-3.64285075
B	3.63850564	7.79923490	-3.63860660
VEC1	12.94100000	0.00000000	0.00000000
VEC2	0.00000000	12.94100000	0.00000000
VEC3	0.00000000	0.00000000	12.94100000

Figure10_b

79

Zn	0.61428565	-1.27937534	-0.52648892
Zn	0.60386565	0.95031652	1.72769122
Zn	-1.63986465	0.95031653	-0.51089712
Zn	-1.62556796	-1.30407120	1.71850956
O	-0.51118378	-0.16936892	0.60156567
O	2.50357990	-0.95090439	-0.20800227
O	2.49596864	0.64430469	1.40477695
O	0.29146519	-0.95090439	-2.41504748
O	-1.32129232	0.64430469	-2.40373602
O	-1.33139586	2.84115184	-0.18119140
O	0.28937697	0.61924839	3.61715915
O	-3.51891781	-0.99431168	1.40254457
O	-3.52860607	0.61924838	-0.19207424
O	-1.30525984	-0.99431168	3.61112949
O	0.27345306	2.84115185	1.41997970
O	-1.29499356	-3.19391881	1.38717584
O	0.30644134	-3.17440520	-0.21793751
O	1.18818610	1.51860358	5.69243504
O	2.75920938	3.10946277	5.73364326
O	1.16065323	4.93037054	-1.07411148
O	2.73963171	4.98339005	-2.65671681
O	4.58044761	-1.83135607	-1.12100721
O	4.62236976	-3.40290579	-2.71133020
O	5.01576264	4.26636083	1.73128093
O	6.30234598	5.98272640	2.68552599
C	2.03715396	2.36500504	-5.18892911
C	0.33512730	0.64109234	-5.16677910
C	-1.36171740	-1.07739242	-5.17808313
C	2.02309249	2.35989449	6.35253470
C	0.33649929	0.65153543	6.38375223

C	-1.36275812	-1.06938883	6.37250201
C	-0.51278291	-0.20403309	5.67735029
C	-0.50706502	-0.19062302	4.18508773
C	-0.51557569	-0.14991028	-2.98030926
C	-0.51118378	5.02561520	0.60156567
C	-1.40844450	5.59123928	1.50088735
C	-1.41712914	-5.95937170	1.50959193
C	-0.56912514	-5.25110089	0.65964010
C	0.28881289	-5.93546962	-0.20026858
C	0.29965336	5.61526660	-0.21113393
C	-4.09470587	-0.19062302	0.60566868
C	1.99531896	5.59673522	-1.91069440
C	1.99944679	-5.94464979	-1.91483172
C	1.14605621	-5.24033995	-1.05948094
C	-0.49089985	-3.75451553	0.58123515
C	-5.59026744	-1.81533147	-1.12709977
C	-5.58895750	-0.10819673	0.60002900
C	-6.30054711	-2.66529283	-1.98131857
C	-6.27917756	-0.96221210	-0.25843809
C	-6.29126932	0.73543560	1.45924318
C	5.24091864	-2.66175276	-1.96645967
C	5.27136061	-0.97403149	-0.25918534
C	5.25932905	0.72602289	1.46092750
C	4.56456339	-0.12961183	0.60085754
C	-0.53227051	3.41190826	0.62270083
C	3.07069181	-0.14991027	0.59773959
C	5.65315475	5.12603715	2.21269997
C	1.19296492	1.50541255	-4.47822596
C	-0.51324374	-0.22263553	-4.47616601
F	-4.22243647	-3.74116692	-3.08659423
F	3.81678793	-6.21143662	-3.73634723
F	3.08173339	-3.85890175	-2.99960430
F	3.12805853	3.45636319	-3.11129967
F	3.84285050	4.19563986	-5.46927102
F	6.36094050	-4.47405770	-3.80906479
H	1.20414141	1.50960845	-3.38443681
H	-2.02867632	-1.74024173	5.82184715
H	-2.07398676	5.03559507	2.16795832
H	-4.49644935	-1.81828356	-1.13894036
H	-2.02125172	-1.74896545	-4.62099892
H	-5.73449229	1.39948979	2.12659565
H	4.70836737	1.38815603	2.13527080
H	1.14962405	-4.14648154	-1.06305697
H	-2.08401342	-5.40725717	2.17800802
H	2.19153421	4.95815181	3.49914670
H	1.96343377	5.40877679	2.93653835
B	3.07714898	-5.26850929	-2.99500936
B	3.11367231	3.45096257	-4.52084603

B	-5.63197141	-3.73737233	-3.07138188
VEC1	12.94100000	0.00000000	0.00000000
VEC2	0.00000000	12.94100000	0.00000000
VEC3	0.00000000	0.00000000	12.94100000

Figure14_a

94

Zn	1.11832588	-1.12823526	1.11702073
O	0.00000000	0.00000000	0.00000000
Zn	-1.11690035	1.12047199	1.12350596
Zn	1.12219394	1.11897262	-1.11971895
Zn	-1.12621595	-1.11650539	-1.12339591
O	0.80213697	-0.81178373	3.00875080
C	0.00305483	-0.00843257	3.58191925
O	-0.79708182	0.79703132	3.01323421
C	3.58191236	-0.01082909	-0.00354043
O	3.00968100	-0.81326198	0.79713438
O	3.01229190	0.79448044	-0.80316691
C	0.00918028	3.58138347	0.00676958
O	0.80842204	3.01007489	-0.79661456
O	-3.00902029	0.80786599	0.80608208
C	-0.00334651	0.00665239	-3.58357973
O	-0.80714607	-0.79471572	-3.01421696
O	0.80107402	0.80531462	-3.01104096
C	-3.58357456	0.00904983	0.00325300
O	-3.01623016	-0.79323645	-0.80105763
O	-0.79311094	3.01114733	0.80787288
C	-0.01001744	-3.58523958	-0.00760262
O	-0.81097469	-3.01160387	-0.80746843
O	0.79382786	-3.01813380	0.79438345
C	-0.00571474	-5.07589619	-0.00231149
H	8.94497845	-1.73802636	1.70882553
C	7.16655713	0.85094258	-0.86564050
B	7.79718631	-3.66246319	3.62865844
C	-4.98356264	0.00836246	-0.00248239
C	5.73601164	-2.56083027	2.53648029
C	5.77726518	-0.86016645	0.84232676
H	7.72757640	1.51545290	-1.52897402
H	5.22941714	1.52528967	-1.53249386
C	5.77612508	0.85328853	-0.86447976
F	6.84439910	-4.39684279	4.36257641
F	9.20666666	-3.67828270	3.64088242
C	-0.01166294	0.00503311	-4.98355409
C	5.07589281	-0.00312662	-0.00963575

H	8.95732133	-0.01270863	-0.00982188
C	7.16777284	-0.86008744	0.83875315
C	7.85120270	-1.72574250	1.69930404
C	7.86344925	-0.00526563	-0.01449110
C	0.04396636	5.19464087	0.04041330
C	-0.85030166	5.77481866	-0.85259583
C	-0.84723648	7.16525704	-0.85046567
C	0.00845759	7.85969098	0.00318860
C	0.86231933	7.16141583	0.85594479
C	0.86141534	5.77106764	0.85597177
O	1.71834058	5.07221542	1.71178622
C	2.56031505	5.72496288	2.55177400
C	2.57629640	7.12441826	2.56679053
C	1.72715998	7.84255923	1.71873767
O	3.30092537	5.09955437	3.29143861
B	3.66187609	7.78298868	3.64993108
F	4.39501394	6.82809579	4.38235721
F	3.67839951	9.19242854	3.66548201
H	-1.52187739	5.23003409	-1.52257081
H	-1.51078171	7.72817984	-1.51316532
H	0.01644550	8.95356000	0.01043066
H	1.73999253	8.93628735	1.73081555
C	1.71261932	-1.72163191	7.84921168
C	0.00000000	0.00000000	7.86346437
C	2.55979964	-2.57324399	7.13250833
C	0.85138892	-0.85585778	7.16678932
C	-0.85186005	0.85631198	7.16756877
C	2.54533688	-2.55869369	5.73304110
C	0.85239936	-0.85686860	5.77627780
C	-0.85326071	0.85772779	5.77713568
C	-0.00028150	0.00027256	5.07590291
O	1.70711511	-1.71605838	5.07888476
O	3.28406975	-3.30129242	5.10889086
H	1.72414872	-1.73319106	8.94297600
H	0.00668056	-0.00671498	8.95733072
B	3.64057594	-3.65967980	7.79293679
H	-1.51371400	1.52164117	7.72936440
H	-1.52183211	1.52981054	5.23120984
F	4.37224537	-4.39518736	6.83929068
F	3.65538739	-3.67456539	9.20240250
O	5.08087469	-1.71831757	1.69890138
O	5.11272795	-3.30251694	3.27685922
C	7.13549312	-2.57630803	2.54837377
H	0.87743529	-5.53817138	-0.41794072
H	-0.88618997	-5.54326193	-0.41792265
H	-0.00467563	-5.43227820	1.01719368
H	-5.33938817	0.00818571	-1.02218243
H	0.86749114	0.44073250	-5.43483467

H	-5.43045891	-0.87367249	0.43191152
H	-0.01380144	-1.01361636	-5.34236976
H	-5.43132480	0.88995991	0.43190797
H	-0.89611036	0.44074462	-5.42435816
H	-4.25842155	2.97668432	-7.39379008
H	-4.50456049	2.84660068	-8.09570539
C	0.20254113	3.78729062	-3.89705119
O	-0.91897523	3.47770644	-3.74599911
O	1.31872693	4.09083824	-4.07738232

Figure14_b

100

Zn	1.11832588	-1.12823526	1.11702073
O	0.00000000	0.00000000	0.00000000
Zn	-1.11690035	1.12047199	1.12350596
Zn	1.12219394	1.11897262	-1.11971895
Zn	-1.12621595	-1.11650539	-1.12339591
O	0.80213697	-0.81178373	3.00875080
C	0.00305483	-0.00843257	3.58191925
O	-0.79708182	0.79703132	3.01323421
C	3.58191236	-0.01082909	-0.00354043
O	3.00968100	-0.81326198	0.79713438
O	3.01229190	0.79448044	-0.80316691
C	0.00918028	3.58138347	0.00676958
O	0.80842204	3.01007489	-0.79661456
O	-3.00902029	0.80786599	0.80608208
C	-0.00334651	0.00665239	-3.58357973
O	-0.80714607	-0.79471572	-3.01421696
O	0.80107402	0.80531462	-3.01104096
C	-3.58357456	0.00904983	0.00325300
O	-3.01623016	-0.79323645	-0.80105763
O	-0.79311094	3.01114733	0.80787288
C	-0.01001744	-3.58523958	-0.00760262
O	-0.81097469	-3.01160387	-0.80746843
O	0.79382786	-3.01813380	0.79438345
C	-0.00571474	-5.07589619	-0.00231149
H	8.94497845	-1.73802636	1.70882553
C	7.16655713	0.85094258	-0.86564050
B	7.79718631	-3.66246319	3.62865844
C	-4.98356264	0.00836246	-0.00248239
C	5.73601164	-2.56083027	2.53648029
C	5.77726518	-0.86016645	0.84232676
H	7.72757640	1.51545290	-1.52897402
B	4.94202950	1.87854011	-1.88364843

C	5.77612508	0.85328853	-0.86447976
F	6.84439910	-4.39684279	4.36257641
F	9.20666666	-3.67828270	3.64088242
C	-0.01166294	0.00503311	-4.98355409
C	5.07589281	-0.00312662	-0.00963575
H	8.95732133	-0.01270863	-0.00982188
C	7.16777284	-0.86008744	0.83875315
C	7.85120270	-1.72574250	1.69930404
C	7.86344925	-0.00526563	-0.01449110
C	0.04396636	5.19464087	0.04041330
C	-0.85030166	5.77481866	-0.85259583
C	-0.84723648	7.16525704	-0.85046567
C	0.00845759	7.85969098	0.00318860
C	0.86231933	7.16141583	0.85594479
C	0.86141534	5.77106764	0.85597177
O	1.71834058	5.07221542	1.71178622
C	2.56031505	5.72496288	2.55177400
C	2.57629640	7.12441826	2.56679053
C	1.72715998	7.84255923	1.71873767
O	3.30092537	5.09955437	3.29143861
B	3.66187609	7.78298868	3.64993108
F	4.39501394	6.82809579	4.38235721
F	3.67839951	9.19242854	3.66548201
B	-1.87492443	4.94364109	-1.87477635
H	-1.51078171	7.72817984	-1.51316532
H	0.01644550	8.95356000	0.01043066
H	1.73999253	8.93628735	1.73081555
C	1.71261932	-1.72163191	7.84921168
C	0.00000000	0.00000000	7.86346437
C	2.55979964	-2.57324399	7.13250833
C	0.85138892	-0.85585778	7.16678932
C	-0.85186005	0.85631198	7.16756877
C	2.54533688	-2.55869369	5.73304110
C	0.85239936	-0.85686860	5.77627780
C	-0.85326071	0.85772779	5.77713568
C	-0.00028150	0.00027256	5.07590291
O	1.70711511	-1.71605838	5.07888476
O	3.28406975	-3.30129242	5.10889086
H	1.72414872	-1.73319106	8.94297600
H	0.00668056	-0.00671498	8.95733072
B	3.64057594	-3.65967980	7.79293679
H	-1.51371400	1.52164117	7.72936440
B	-1.87327962	1.88310386	4.94423333
F	4.37224537	-4.39518736	6.83929068
F	3.65538739	-3.67456539	9.20240250
O	5.08087469	-1.71831757	1.69890138
O	5.11272795	-3.30251694	3.27685922
C	7.13549312	-2.57630803	2.54837377

H	-0.00467563	-5.43227820	1.01719368
H	-0.88618997	-5.54326193	-0.41792265
H	0.87743529	-5.53817138	-0.41794072
H	-5.33938817	0.00818571	-1.02218243
H	0.86749114	0.44073250	-5.43483467
H	-5.43045891	-0.87367249	0.43191152
H	-0.01380144	-1.01361636	-5.34236976
H	-5.43132480	0.88995991	0.43190797
H	-0.89611036	0.44074462	-5.42435816
F	4.65550053	3.19774429	-1.47658399
F	4.43796793	1.39755669	-3.10948564
F	-3.17315302	1.45842343	4.60061191
F	-1.45644432	3.19410813	4.63501477
F	-1.56620905	4.83400624	-3.24618985
F	-3.06686494	4.38119313	-1.37374730
H	3.67384893	0.57008911	6.69744952
H	3.15490186	1.02283000	6.38761639
C	3.31616055	2.08799465	3.22104261
O	4.25647094	2.05062090	2.52180965
O	2.37023398	2.09230719	3.91343241

Figure14_c

94

Zn	1.11832588	-1.12823526	1.11702073
O	0.00000000	0.00000000	0.00000000
Zn	-1.11690035	1.12047199	1.12350596
Zn	1.12219394	1.11897262	-1.11971895
Zn	-1.12621595	-1.11650539	-1.12339591
O	0.80213697	-0.81178373	3.00875080
C	0.00305483	-0.00843257	3.58191925
O	-0.79708182	0.79703132	3.01323421
C	3.58191236	-0.01082909	-0.00354043
O	3.00968100	-0.81326198	0.79713438
O	3.01229190	0.79448044	-0.80316691
C	0.00918028	3.58138347	0.00676958
O	0.80842204	3.01007489	-0.79661456
O	-3.00902029	0.80786599	0.80608208
C	-0.00334651	0.00665239	-3.58357973
O	-0.80714607	-0.79471572	-3.01421696
O	0.80107402	0.80531462	-3.01104096
C	-3.58357456	0.00904983	0.00325300
O	-3.01623016	-0.79323645	-0.80105763
O	-0.79311094	3.01114733	0.80787288
C	-0.01001744	-3.58523958	-0.00760262

O	-0.81097469	-3.01160387	-0.80746843
O	0.79382786	-3.01813380	0.79438345
C	-0.00571474	-5.07589619	-0.00231149
H	8.94497845	-1.73802636	1.70882553
C	7.16655713	0.85094258	-0.86564050
H	7.56929725	-3.28838819	3.25660525
C	-4.98356264	0.00836246	-0.00248239
C	5.73601164	-2.56083027	2.53648029
C	5.77726518	-0.86016645	0.84232676
H	7.72757640	1.51545290	-1.52897402
B	4.94202950	1.87854011	-1.88364843
C	5.77612508	0.85328853	-0.86447976
H	2.35711156	2.84980607	4.46904492
F	-3.17315302	1.45842343	4.60061191
C	-0.01166294	0.00503311	-4.98355409
C	5.07589281	-0.00312662	-0.00963575
H	8.95732133	-0.01270863	-0.00982188
C	7.16777284	-0.86008744	0.83875315
C	7.85120270	-1.72574250	1.69930404
C	7.86344925	-0.00526563	-0.01449110
C	0.04396636	5.19464087	0.04041330
C	-0.85030166	5.77481866	-0.85259583
C	-0.84723648	7.16525704	-0.85046567
C	0.00845759	7.85969098	0.00318860
C	0.86231933	7.16141583	0.85594479
C	0.86141534	5.77106764	0.85597177
O	1.71834058	5.07221542	1.71178622
C	2.56031505	5.72496288	2.55177400
C	2.57629640	7.12441826	2.56679053
C	1.72715998	7.84255923	1.71873767
O	3.30092537	5.09955437	3.29143861
H	3.28789632	7.55611265	3.27679159
F	-3.06686494	4.38119313	-1.37374730
H	2.04506554	2.28570622	4.86343184
B	-1.87492443	4.94364109	-1.87477635
H	-1.51078171	7.72817984	-1.51316532
H	0.01644550	8.95356000	0.01043066
H	1.73999253	8.93628735	1.73081555
C	1.71261932	-1.72163191	7.84921168
C	0.00000000	0.00000000	7.86346437
C	2.55979964	-2.57324399	7.13250833
C	0.85138892	-0.85585778	7.16678932
C	-0.85186005	0.85631198	7.16756877
C	2.54533688	-2.55869369	5.73304110
C	0.85239936	-0.85686860	5.77627780
C	-0.85326071	0.85772779	5.77713568
C	-0.00028150	0.00027256	5.07590291
O	1.70711511	-1.71605838	5.07888476

O	3.28406975	-3.30129242	5.10889086
H	1.72414872	-1.73319106	8.94297600
H	0.00668056	-0.00671498	8.95733072
H	3.26835343	-3.28550813	7.56548331
H	-1.51371400	1.52164117	7.72936440
B	-1.87327962	1.88310386	4.94423333
F	-1.45644432	3.19410813	4.63501477
F	-1.56620905	4.83400624	-3.24618985
O	5.08087469	-1.71831757	1.69890138
O	5.11272795	-3.30251694	3.27685922
C	7.13549312	-2.57630803	2.54837377
H	-0.00467563	-5.43227820	1.01719368
H	-0.88618997	-5.54326193	-0.41792265
H	0.87743529	-5.53817138	-0.41794072
H	-5.33938817	0.00818571	-1.02218243
H	0.86749114	0.44073250	-5.43483467
H	-5.43045891	-0.87367249	0.43191152
H	-0.01380144	-1.01361636	-5.34236976
H	-5.43132480	0.88995991	0.43190797
H	-0.89611036	0.44074462	-5.42435816
F	4.65550053	3.19774429	-1.47658399
F	4.43796793	1.39755669	-3.10948564
C	4.74661749	0.28278808	4.78233140
O	5.38017997	0.43711011	3.81058865
O	4.12575807	0.13828176	5.76426520

Figure17

103

Zn	1.11832588	-1.12823526	1.11702073
O	0.00000000	0.00000000	0.00000000
Zn	-1.11690035	1.12047199	1.12350596
Zn	1.12219394	1.11897262	-1.11971895
Zn	-1.12621595	-1.11650539	-1.12339591
O	0.80213697	-0.81178373	3.00875080
C	0.00305483	-0.00843257	3.58191925
O	-0.79708182	0.79703132	3.01323421
C	3.58191236	-0.01082909	-0.00354043
O	3.00968100	-0.81326198	0.79713438
O	3.01229190	0.79448044	-0.80316691
C	0.00918028	3.58138347	0.00676958
O	0.80842204	3.01007489	-0.79661456
O	-3.00902029	0.80786599	0.80608208
C	-0.00334651	0.00665239	-3.58357973
O	-0.80714607	-0.79471572	-3.01421696

O	0.80107402	0.80531462	-3.01104096
C	-3.58357456	0.00904983	0.00325300
O	-3.01623016	-0.79323645	-0.80105763
O	-0.79311094	3.01114733	0.80787288
C	-0.01001744	-3.58523958	-0.00760262
O	-0.81097469	-3.01160387	-0.80746843
O	0.79382786	-3.01813380	0.79438345
C	-0.00571474	-5.07589619	-0.00231149
H	8.94497845	-1.73802636	1.70882553
C	7.16655713	0.85094258	-0.86564050
C	7.73346078	-3.55785911	3.52461972
C	-4.98356264	0.00836246	-0.00248239
C	5.73601164	-2.56083027	2.53648029
C	5.77726518	-0.86016645	0.84232676
H	7.72757640	1.51545290	-1.52897402
H	5.22941714	1.52528967	-1.53249386
C	5.77612508	0.85328853	-0.86447976
F	6.18673169	-4.01602009	5.52094577
H	-0.89611036	0.44074462	-5.42435816
C	-0.01166294	0.00503311	-4.98355409
C	5.07589281	-0.00312662	-0.00963575
H	8.95732133	-0.01270863	-0.00982188
C	7.16777284	-0.86008744	0.83875315
C	7.85120270	-1.72574250	1.69930404
C	7.86344925	-0.00526563	-0.01449110
C	0.04396636	5.19464087	0.04041330
C	-0.85030166	5.77481866	-0.85259583
C	-0.84723648	7.16525704	-0.85046567
C	0.00845759	7.85969098	0.00318860
C	0.86231933	7.16141583	0.85594479
C	0.86141534	5.77106764	0.85597177
O	1.71834058	5.07221542	1.71178622
C	2.56031505	5.72496288	2.55177400
C	2.57629640	7.12441826	2.56679053
C	1.72715998	7.84255923	1.71873767
O	3.30092537	5.09955437	3.29143861
C	3.55718551	7.71947780	3.54547572
H	3.56871125	-4.46551083	7.11142612
H	3.20536258	-3.85885368	8.73828478
H	-1.52187739	5.23003409	-1.52257081
H	-1.51078171	7.72817984	-1.51316532
H	0.01644550	8.95356000	0.01043066
H	1.73999253	8.93628735	1.73081555
C	1.71261932	-1.72163191	7.84921168
C	0.00000000	0.00000000	7.86346437
C	2.55979964	-2.57324399	7.13250833
C	0.85138892	-0.85585778	7.16678932
C	-0.85186005	0.85631198	7.16756877

C	2.54533688	-2.55869369	5.73304110
C	0.85239936	-0.85686860	5.77627780
C	-0.85326071	0.85772779	5.77713568
C	-0.00028150	0.00027256	5.07590291
O	1.70711511	-1.71605838	5.07888476
O	3.28406975	-3.30129242	5.10889086
H	1.72414872	-1.73319106	8.94297600
H	0.00668056	-0.00671498	8.95733072
C	3.53648987	-3.55504868	7.72933306
H	-1.51371400	1.52164117	7.72936440
H	-1.52183211	1.52981054	5.23120984
H	8.33684340	-3.02058490	4.27332473
H	8.39306038	-4.26450035	2.99773543
O	5.08087469	-1.71831757	1.69890138
O	5.11272795	-3.30251694	3.27685922
C	7.13549312	-2.57630803	2.54837377
H	0.87743529	-5.53817138	-0.41794072
H	-0.88618997	-5.54326193	-0.41792265
H	-0.00467563	-5.43227820	1.01719368
H	-5.33938817	0.00818571	-1.02218243
H	0.86749114	0.44073250	-5.43483467
H	-5.43045891	-0.87367249	0.43191152
H	-0.01380144	-1.01361636	-5.34236976
H	-5.43132480	0.88995991	0.43190797
B	6.45882376	-4.30393472	4.20872140
F	6.14646445	-5.57412199	3.81517588
F	5.37992157	-1.78076178	7.48358755
F	5.94667886	-3.75164753	8.49721973
B	5.01811061	-3.01400360	7.86677065
F	3.38215736	6.23581028	5.62611031
F	5.39275324	6.17684181	4.48965992
B	4.07700750	6.51563685	4.48847661
H	3.07518463	8.52165655	4.12534494
H	4.39940496	8.16692322	2.99057428
H	2.72512951	2.04510684	3.62475304
H	2.74723214	1.29459947	3.54074675
C	5.47018927	3.14129219	1.69125369
O	5.14104097	3.97623518	0.93803532
O	5.80820447	2.29792763	2.42822450

FigureS1_CBF2

24

C	0.55186846	-1.68406980	0.00000000
C	-1.90865255	-1.22778511	0.00000000

C 1.84567584 -1.23260413 0.00000000
C -0.56553751 -0.79626727 0.00000000
C -2.94248686 -0.30558128 0.00000000
C 2.11830683 0.20723186 0.00000000
C -0.29624392 0.58937088 0.00000000
C -2.64988260 1.06890826 0.00000000
C -1.33640368 1.52269848 0.00000000
O 0.97726463 1.06574486 0.00000000
O 3.19434895 0.75083680 0.00000000
H 0.36087964 -2.76075424 0.00000000
H -2.11720802 -2.29911935 0.00000000
B 3.01375519 -2.25891317 0.00000000
H -3.97927948 -0.64171345 0.00000000
H -3.46420753 1.79496074 0.00000000
H -1.09272945 2.58522933 0.00000000
F 2.72317686 -3.57219154 0.00000000
F 4.30075431 -1.92472277 0.00000000
H 3.71040124 0.70439814 -2.59836890
H 3.64910618 0.66672274 -3.35112939
C 4.22992688 -2.21341353 -4.11317570
O 4.26120904 -1.74478046 -5.18507581
O 4.19885418 -2.68652369 -3.04267108

FigureS1_IRMOCCBF2

94

Zn 1.11832588 -1.12823526 1.11702073
O 0.00000000 0.00000000 0.00000000
Zn -1.11690035 1.12047199 1.12350596
Zn 1.12219394 1.11897262 -1.11971895
Zn -1.12621595 -1.11650539 -1.12339591
O 0.80213697 -0.81178373 3.00875080
C 0.00305483 -0.00843257 3.58191925
O -0.79708182 0.79703132 3.01323421
C 3.58191236 -0.01082909 -0.00354043
O 3.00968100 -0.81326198 0.79713438
O 3.01229190 0.79448044 -0.80316691
C 0.00918028 3.58138347 0.00676958
O 0.80842204 3.01007489 -0.79661456
O -3.00902029 0.80786599 0.80608208
C -0.00334651 0.00665239 -3.58357973
O -0.80714607 -0.79471572 -3.01421696
O 0.80107402 0.80531462 -3.01104096
C -3.58357456 0.00904983 0.00325300
O -3.01623016 -0.79323645 -0.80105763

O	-0.79311094	3.01114733	0.80787288
C	-0.01001744	-3.58523958	-0.00760262
O	-0.81097469	-3.01160387	-0.80746843
O	0.79382786	-3.01813380	0.79438345
C	-0.00571474	-5.07589619	-0.00231149
H	8.94497845	-1.73802636	1.70882553
C	7.16655713	0.85094258	-0.86564050
B	7.79718631	-3.66246319	3.62865844
C	-4.98356264	0.00836246	-0.00248239
C	5.73601164	-2.56083027	2.53648029
C	5.77726518	-0.86016645	0.84232676
H	7.72757640	1.51545290	-1.52897402
H	5.22941714	1.52528967	-1.53249386
C	5.77612508	0.85328853	-0.86447976
F	6.84439910	-4.39684279	4.36257641
F	9.20666666	-3.67828270	3.64088242
C	-0.01166294	0.00503311	-4.98355409
C	5.07589281	-0.00312662	-0.00963575
H	8.95732133	-0.01270863	-0.00982188
C	7.16777284	-0.86008744	0.83875315
C	7.85120270	-1.72574250	1.69930404
C	7.86344925	-0.00526563	-0.01449110
C	0.04396636	5.19464087	0.04041330
C	-0.85030166	5.77481866	-0.85259583
C	-0.84723648	7.16525704	-0.85046567
C	0.00845759	7.85969098	0.00318860
C	0.86231933	7.16141583	0.85594479
C	0.86141534	5.77106764	0.85597177
O	1.71834058	5.07221542	1.71178622
C	2.56031505	5.72496288	2.55177400
C	2.57629640	7.12441826	2.56679053
C	1.72715998	7.84255923	1.71873767
O	3.30092537	5.09955437	3.29143861
B	3.66187609	7.78298868	3.64993108
F	4.39501394	6.82809579	4.38235721
F	3.67839951	9.19242854	3.66548201
H	-1.52187739	5.23003409	-1.52257081
H	-1.51078171	7.72817984	-1.51316532
H	0.01644550	8.95356000	0.01043066
H	1.73999253	8.93628735	1.73081555
C	1.71261932	-1.72163191	7.84921168
C	0.00000000	0.00000000	7.86346437
C	2.55979964	-2.57324399	7.13250833
C	0.85138892	-0.85585778	7.16678932
C	-0.85186005	0.85631198	7.16756877
C	2.54533688	-2.55869369	5.73304110
C	0.85239936	-0.85686860	5.77627780
C	-0.85326071	0.85772779	5.77713568

C	-0.00028150	0.00027256	5.07590291
O	1.70711511	-1.71605838	5.07888476
O	3.28406975	-3.30129242	5.10889086
H	1.72414872	-1.73319106	8.94297600
H	0.00668056	-0.00671498	8.95733072
B	3.64057594	-3.65967980	7.79293679
H	-1.51371400	1.52164117	7.72936440
H	-1.52183211	1.52981054	5.23120984
F	4.37224537	-4.39518736	6.83929068
F	3.65538739	-3.67456539	9.20240250
O	5.08087469	-1.71831757	1.69890138
O	5.11272795	-3.30251694	3.27685922
C	7.13549312	-2.57630803	2.54837377
H	0.87743529	-5.53817138	-0.41794072
H	-0.88618997	-5.54326193	-0.41792265
H	-0.00467563	-5.43227820	1.01719368
H	-5.33938817	0.00818571	-1.02218243
H	0.86749114	0.44073250	-5.43483467
H	-5.43045891	-0.87367249	0.43191152
H	-0.01380144	-1.01361636	-5.34236976
H	-5.43132480	0.88995991	0.43190797
H	-0.89611036	0.44074462	-5.42435816
H	-4.25842155	2.97668432	-7.39379008
H	-4.50456049	2.84660068	-8.09570539
C	0.20254113	3.78729062	-3.89705119
O	-0.91897523	3.47770644	-3.74599911
O	1.31872693	4.09083824	-4.07738232

FigureS5_a1

94

Zn	1.11832588	-1.12823526	1.11702073
O	0.00000000	0.00000000	0.00000000
Zn	-1.11690035	1.12047199	1.12350596
Zn	1.12219395	1.11897262	-1.11971895
Zn	-1.12621595	-1.11650539	-1.12339591
O	0.80213697	-0.81178373	3.00875080
C	0.00305483	-0.00843257	3.58191925
O	-0.79708182	0.79703132	3.01323421
C	3.58191236	-0.01082909	-0.00354043
O	3.00968100	-0.81326198	0.79713438
O	3.01229190	0.79448044	-0.80316691
C	0.00918028	3.58138347	0.00676958
O	0.80842204	3.01007489	-0.79661456
O	-3.00902029	0.80786599	0.80608208

C	-0.00334651	0.00665239	-3.58357973
O	-0.80714607	-0.79471572	-3.01421696
O	0.80107402	0.80531462	-3.01104096
C	-3.58357456	0.00904983	0.00325300
O	-3.01623017	-0.79323645	-0.80105763
O	-0.79311094	3.01114733	0.80787288
C	-0.01001744	-3.58523958	-0.00760262
O	-0.81097469	-3.01160387	-0.80746843
O	0.79382786	-3.01813380	0.79438345
C	-0.00571474	-5.07589619	-0.00231149
H	8.94497845	-1.73802636	1.70882553
C	7.16655713	0.85094258	-0.86564050
B	7.79718631	-3.66246319	3.62865844
C	-4.98356264	0.00836246	-0.00248239
C	5.73601164	-2.56083027	2.53648029
C	5.77726518	-0.86016645	0.84232676
H	7.72757640	1.51545290	-1.52897402
H	5.22941714	1.52528967	-1.53249386
C	5.77612508	0.85328853	-0.86447976
F	6.84439910	-4.39684279	4.36257641
F	9.20666666	-3.67828270	3.64088242
C	-0.01166294	0.00503311	-4.98355409
C	5.07589281	-0.00312662	-0.00963575
H	8.95732133	-0.01270863	-0.00982188
C	7.16777284	-0.86008744	0.83875315
C	7.85120270	-1.72574250	1.69930405
C	7.86344925	-0.00526563	-0.01449110
C	0.04396636	5.19464087	0.04041330
C	-0.85030166	5.77481866	-0.85259583
C	-0.84723648	7.16525704	-0.85046567
C	0.00845759	7.85969098	0.00318860
C	0.86231933	7.16141583	0.85594479
C	0.86141534	5.77106764	0.85597177
O	1.71834058	5.07221542	1.71178622
C	2.56031505	5.72496288	2.55177400
C	2.57629640	7.12441826	2.56679053
C	1.72715998	7.84255923	1.71873767
O	3.30092537	5.09955437	3.29143861
B	3.66187609	7.78298868	3.64993108
F	4.39501394	6.82809579	4.38235721
F	3.67839951	9.19242854	3.66548201
H	-1.52187739	5.23003409	-1.52257081
H	-1.51078171	7.72817984	-1.51316532
H	0.01644550	8.95356000	0.01043066
H	1.73999253	8.93628735	1.73081555
C	1.71261932	-1.72163191	7.84921168
C	0.00000000	0.00000000	7.86346437
C	2.55979964	-2.57324399	7.13250833

C	0.85138892	-0.85585778	7.16678932
C	-0.85186005	0.85631198	7.16756877
C	2.54533688	-2.55869369	5.73304110
C	0.85239936	-0.85686860	5.77627780
C	-0.85326071	0.85772779	5.77713568
C	-0.00028150	0.00027256	5.07590291
O	1.70711511	-1.71605838	5.07888476
O	3.28406975	-3.30129242	5.10889086
H	1.72414872	-1.73319106	8.94297600
H	0.00668056	-0.00671498	8.95733072
B	3.64057594	-3.65967980	7.79293679
H	-1.51371401	1.52164117	7.72936440
H	-1.52183211	1.52981054	5.23120984
F	4.37224537	-4.39518736	6.83929068
F	3.65538739	-3.67456539	9.20240250
O	5.08087469	-1.71831757	1.69890139
O	5.11272795	-3.30251694	3.27685922
C	7.13549312	-2.57630803	2.54837377
H	0.87743529	-5.53817138	-0.41794072
H	-0.88618997	-5.54326193	-0.41792265
H	-0.00467563	-5.43227820	1.01719368
H	-5.33938817	0.00818571	-1.02218243
H	0.86749114	0.44073250	-5.43483467
H	-5.43045891	-0.87367249	0.43191152
H	-0.01380144	-1.01361636	-5.34236976
H	-5.43132480	0.88995991	0.43190797
H	-0.89611036	0.44074462	-5.42435816
H	-2.73049844	6.00647153	-4.03506417
H	-1.99657974	5.83125372	-4.09542025
C	1.24400403	5.74425948	-2.84530921
O	0.91220777	4.99073146	-3.87871519
O	2.47601841	5.99700813	-2.45096957

FigureS5_a2

94

Zn	1.11832588	-1.12823526	1.11702073
O	0.00000000	0.00000000	0.00000000
Zn	-1.11690035	1.12047199	1.12350596
Zn	1.12219395	1.11897262	-1.11971895
Zn	-1.12621595	-1.11650539	-1.12339591
O	0.80213697	-0.81178373	3.00875080
C	0.00305483	-0.00843257	3.58191925
O	-0.79708182	0.79703132	3.01323421
C	3.58191236	-0.01082909	-0.00354043

O	3.00968100	-0.81326198	0.79713438
O	3.01229190	0.79448044	-0.80316691
C	0.00918028	3.58138347	0.00676958
O	0.80842204	3.01007489	-0.79661456
O	-3.00902029	0.80786599	0.80608208
C	-0.00334651	0.00665239	-3.58357973
O	-0.80714607	-0.79471572	-3.01421696
O	0.80107402	0.80531462	-3.01104096
C	-3.58357456	0.00904983	0.00325300
O	-3.01623017	-0.79323645	-0.80105763
O	-0.79311094	3.01114733	0.80787288
C	-0.01001744	-3.58523958	-0.00760262
O	-0.81097469	-3.01160387	-0.80746843
O	0.79382786	-3.01813380	0.79438345
C	-0.00571474	-5.07589619	-0.00231149
H	8.94497845	-1.73802636	1.70882553
C	7.16655713	0.85094258	-0.86564050
B	7.79718631	-3.66246319	3.62865844
C	-4.98356264	0.00836246	-0.00248239
C	5.73601164	-2.56083027	2.53648029
C	5.77726518	-0.86016645	0.84232676
H	7.72757640	1.51545290	-1.52897402
H	5.22941714	1.52528967	-1.53249386
C	5.77612508	0.85328853	-0.86447976
F	6.84439910	-4.39684279	4.36257641
F	9.20666666	-3.67828270	3.64088242
C	-0.01166294	0.00503311	-4.98355409
C	5.07589281	-0.00312662	-0.00963575
H	8.95732133	-0.01270863	-0.00982188
C	7.16777284	-0.86008744	0.83875315
C	7.85120270	-1.72574250	1.69930405
C	7.86344925	-0.00526563	-0.01449110
C	0.04396636	5.19464087	0.04041330
C	-0.85030166	5.77481866	-0.85259583
C	-0.84723648	7.16525704	-0.85046567
C	0.00845759	7.85969098	0.00318860
C	0.86231933	7.16141583	0.85594479
C	0.86141534	5.77106764	0.85597177
O	1.71834058	5.07221542	1.71178622
C	2.56031505	5.72496288	2.55177400
C	2.57629640	7.12441826	2.56679053
C	1.72715998	7.84255923	1.71873767
O	3.30092537	5.09955437	3.29143861
B	3.66187609	7.78298868	3.64993108
F	4.39501394	6.82809579	4.38235721
F	3.67839951	9.19242854	3.66548201
H	-1.52187739	5.23003409	-1.52257081
H	-1.51078171	7.72817984	-1.51316532

H	0.01644550	8.95356000	0.01043066
H	1.73999253	8.93628735	1.73081555
C	1.71261932	-1.72163191	7.84921168
C	0.00000000	0.00000000	7.86346437
C	2.55979964	-2.57324399	7.13250833
C	0.85138892	-0.85585778	7.16678932
C	-0.85186005	0.85631198	7.16756877
C	2.54533688	-2.55869369	5.73304110
C	0.85239936	-0.85686860	5.77627780
C	-0.85326071	0.85772779	5.77713568
C	-0.00028150	0.00027256	5.07590291
O	1.70711511	-1.71605838	5.07888476
O	3.28406975	-3.30129242	5.10889086
H	1.72414872	-1.73319106	8.94297600
H	0.00668056	-0.00671498	8.95733072
B	3.64057594	-3.65967980	7.79293679
H	-1.51371401	1.52164117	7.72936440
H	-1.52183211	1.52981054	5.23120984
F	4.37224537	-4.39518736	6.83929068
F	3.65538739	-3.67456539	9.20240250
O	5.08087469	-1.71831757	1.69890139
O	5.11272795	-3.30251694	3.27685922
C	7.13549312	-2.57630803	2.54837377
H	0.87743529	-5.53817138	-0.41794072
H	-0.88618997	-5.54326193	-0.41792265
H	-0.00467563	-5.43227820	1.01719368
H	-5.33938817	0.00818571	-1.02218243
H	0.86749114	0.44073250	-5.43483467
H	-5.43045891	-0.87367249	0.43191152
H	-0.01380144	-1.01361636	-5.34236976
H	-5.43132480	0.88995991	0.43190797
H	-0.89611036	0.44074462	-5.42435816
H	-2.69511614	5.89621390	-4.13509662
H	-2.24800687	6.04218884	-4.41780917
C	1.50588015	5.51802266	-3.02978330
O	0.70565450	4.98154328	-3.71971965
O	2.31081017	6.07186956	-2.37960713

FigureS5_a3

94
Frame 7: Geometry 8, SCF Bond Energy: -22.23468 Hartree
Zn 1.118325877 -1.128235255 1.117020731
O 0.0 0.0 0.0
Zn -1.116900352 1.12047199 1.123505959
Zn 1.122193945 1.118972622 -1.119718947

Zn -1.126215951 -1.116505389 -1.12339591
O 0.8021369693 -0.8117837309 3.008750804
C 0.003054830187 -0.008432567086999999 3.581919254
O -0.7970818184999999 0.7970313188999999 3.013234213
C 3.581912359 -0.01082908697 -0.003540432186
O 3.009680999 -0.8132619803999999 0.7971343812999999
O 3.012291901999999 0.7944804435999999 -0.8031669120999999
C 0.009180282035000001 3.581383465 0.006769578978999999
O 0.8084220440999999 3.010074891 -0.7966145626999999
O -3.009020291 0.8078659864999999 0.8060820827999999
C -0.003346512798000001 0.006652391428999999 -3.583579729000001
O -0.8071460657 -0.7947157234999999 -3.014216963
O 0.8010740201999999 0.8053146168000001 -3.011040958
C -3.583574564 0.009049827377000001 0.003253003893
O -3.016230165 -0.7932364457 -0.8010576277999999
O -0.7931109439999999 3.011147329 0.8078728793999999
C -0.01001744098 -3.585239577 -0.00760262035
O -0.8109746898 -3.011603871 -0.8074684318999998
O 0.7938278576999999 -3.018133795999999 0.794383446
C -0.005714743448 -5.075896192 -0.002311488867
H 8.944978446999999 -1.73802636 1.708825535
C 7.166557128 0.8509425830999998 -0.8656405017999999
B 7.797186308999999 -3.662463194 3.628658441999999
C -4.98356264 0.008362464348999999 -0.002482391202
C 5.736011636999999 -2.560830268 2.536480292999999
C 5.777265182999999 -0.8601664526 0.8423267605
H 7.727576396999999 1.515452898 -1.528974018
H 5.229417135 1.525289674 -1.532493864
C 5.776125075 0.8532885278 -0.8644797624
F 6.844399095999999 -4.396842793999999 4.362576411
F 9.206666662 -3.678282702 3.640882422
C -0.01166293599 0.005033113740999999 -4.983554086999999
C 5.075892805999999 -0.003126622774 -0.009635751917999999
H 8.957321327999999 -0.01270862644 -0.009821881387999998
C 7.167772842999999 -0.8600874402999998 0.8387531465999999
C 7.851202703 -1.725742503 1.699304045
C 7.863449254999999 -0.005265627748 -0.01449109999
C 0.04396635773 5.194640869999999 0.04041330098999999
C -0.8503016577999998 5.774818662 -0.8525958258999999
C -0.8472364843 7.165257035999999 -0.8504656691999998
C 0.0084575933 7.859690976999999 0.003188601708999999
C 0.8623193297 7.161415834000001 0.8559447949
C 0.8614153359999999 5.771067636999998 0.8559717714999999
O 1.718340578 5.072215416 1.711786215
C 2.560315049 5.724962876 2.551773997999999
C 2.576296403 7.12441826 2.566790525999999
C 1.727159976 7.842559230999999 1.718737673
O 3.300925372 5.099554367 3.291438609

B 3.661876094 7.782988681000001 3.649931079999999
F 4.395013939 6.828095789999999 4.382357207
F 3.678399509 9.192428536999998 3.665482008
H -1.52187739 5.230034086 -1.522570815
H -1.510781707 7.728179841999999 -1.513165323
H 0.01644550436 8.95356 0.01043066254
H 1.739992534 8.936287349000001 1.730815553
C 1.712619317 -1.721631909 7.849211683999999
C 0.0 0.0 7.863464373999999
C 2.559799639 -2.573243995 7.132508334999999
C 0.8513889166999999 -0.855857778 7.166789323999999
C -0.8518600472 0.8563119774999999 7.167568769999999
C 2.545336882 -2.558693685000001 5.733041101
C 0.8523993626999999 -0.856868599 5.7762778
C -0.8532607063 0.8577277862999999 5.777135683999999
C -0.0002814954319999999 0.000272557141 5.075902906999999
O 1.707115111 -1.71605838 5.078884757999999
O 3.284069746 -3.301292418 5.108890862
H 1.72414872 -1.733191061 8.942975999
H 0.006680556007 -0.006714976792999999 8.957330720999998
B 3.640575938 -3.659679796 7.792936794999999
H -1.513714005 1.521641171 7.729364400999998
H -1.521832107 1.529810538 5.231209839
F 4.372245368999999 -4.395187360999999 6.839290683999999
F 3.655387387 -3.674565386 9.2024025
O 5.080874688 -1.718317566 1.698901385
O 5.112727950999999 -3.302516944 3.276859219
C 7.135493120999999 -2.576308034999999 2.548373772
H 0.8774352939 -5.538171384 -0.4179407209
H -0.8861899740999999 -5.543261926 -0.4179226517999999
H -0.004675634316 -5.432278195 1.017193679
H -5.339388166 0.008185713854999999 -1.022182427
H 0.8674911352999999 0.4407325033999999 -5.434834666
H -5.430458905999999 -0.8736724881 0.4319115204
H -0.01380144482 -1.013616356 -5.342369757
H -5.431324795 0.8899599141999999 0.4319079731
H -0.8961103623999999 0.4407446211 -5.424358162
H -2.501352412446134 5.819723865990614 -4.124939180423858
H -2.480124445960452 6.120764524823906 -4.45211598140008
C 1.504592140119092 5.505933092031738 -3.036503686934386
O 0.7321209920808048 4.979472080594086 -3.700753384072784
O 2.29689550888802 6.060037897503809 -2.394248380299257

FigureS5_a4

Frame 9: Geometry 10, SCF Bond Energy: -22.33717 Hartree
Zn 1.118325877 -1.128235255 1.117020731
O 0.0 0.0 0.0
Zn -1.116900352 1.12047199 1.123505959
Zn 1.122193945 1.118972622 -1.119718947
Zn -1.126215951 -1.116505389 -1.12339591
O 0.8021369693 -0.8117837309 3.008750804
C 0.003054830187 -0.008432567086999999 3.581919254
O -0.7970818184999999 0.7970313188999999 3.013234213
C 3.581912359 -0.01082908697 -0.003540432186
O 3.009680999 -0.8132619803999999 0.7971343812999999
O 3.012291901999999 0.7944804435999999 -0.8031669120999999
C 0.009180282035000001 3.581383465 0.006769578978999999
O 0.8084220440999999 3.010074891 -0.7966145626999999
O -3.009020291 0.8078659864999999 0.8060820827999999
C -0.003346512798000001 0.006652391428999999 -3.583579729000001
O -0.8071460657 -0.7947157234999999 -3.014216963
O 0.8010740201999999 0.8053146168000001 -3.011040958
C -3.583574564 0.009049827377000001 0.003253003893
O -3.016230165 -0.7932364457 -0.8010576277999999
O -0.7931109439999999 3.011147329 0.8078728793999999
C -0.01001744098 -3.585239577 -0.00760262035
O -0.8109746898 -3.011603871 -0.8074684318999998
O 0.7938278576999999 -3.018133795999999 0.794383446
C -0.005714743448 -5.075896192 -0.002311488867
H 8.944978446999999 -1.73802636 1.708825535
C 7.166557128 0.8509425830999998 -0.8656405017999999
B 7.797186308999999 -3.662463194 3.628658441999999
C -4.98356264 0.008362464348999999 -0.002482391202
C 5.736011636999999 -2.560830268 2.536480292999999
C 5.777265182999999 -0.8601664526 0.8423267605
H 7.727576396999999 1.515452898 -1.528974018
H 5.229417135 1.525289674 -1.532493864
C 5.776125075 0.8532885278 -0.8644797624
F 6.844399095999999 -4.396842793999999 4.362576411
F 9.206666662 -3.678282702 3.640882422
C -0.01166293599 0.005033113740999999 -4.983554086999999
C 5.075892805999999 -0.003126622774 -0.009635751917999999
H 8.957321327999999 -0.01270862644 -0.009821881387999998
C 7.167772842999999 -0.8600874402999998 0.8387531465999999
C 7.851202703 -1.725742503 1.699304045
C 7.863449254999999 -0.005265627748 -0.01449109999
C 0.04396635773 5.194640869999999 0.04041330098999999
C -0.8503016577999998 5.774818662 -0.8525958258999999
C -0.8472364843 7.165257035999999 -0.8504656691999998
C 0.0084575933 7.859690976999999 0.003188601708999999
C 0.8623193297 7.161415834000001 0.8559447949
C 0.8614153359999999 5.771067636999998 0.8559717714999999

O 1.718340578 5.072215416 1.711786215
C 2.560315049 5.724962876 2.551773997999999
C 2.576296403 7.12441826 2.566790525999999
C 1.727159976 7.842559230999999 1.718737673
O 3.300925372 5.099554367 3.291438609
B 3.661876094 7.782988681000001 3.649931079999999
F 4.395013939 6.828095789999999 4.382357207
F 3.678399509 9.192428536999998 3.665482008
H -1.52187739 5.230034086 -1.522570815
H -1.510781707 7.728179841999999 -1.513165323
H 0.01644550436 8.95356 0.01043066254
H 1.739992534 8.936287349000001 1.730815553
C 1.712619317 -1.721631909 7.849211683999999
C 0.0 0.0 7.863464373999999
C 2.559799639 -2.573243995 7.132508334999999
C 0.8513889166999999 -0.855857778 7.166789323999999
C -0.8518600472 0.8563119774999999 7.167568769999999
C 2.545336882 -2.558693685000001 5.733041101
C 0.8523993626999999 -0.856868599 5.7762778
C -0.8532607063 0.8577277862999999 5.777135683999999
C -0.0002814954319999999 0.000272557141 5.075902906999999
O 1.707115111 -1.71605838 5.078884757999999
O 3.284069746 -3.301292418 5.108890862
H 1.72414872 -1.733191061 8.942975999
H 0.006680556007 -0.006714976792999999 8.957330720999998
B 3.640575938 -3.659679796 7.792936794999999
H -1.513714005 1.521641171 7.729364400999998
H -1.521832107 1.529810538 5.231209839
F 4.372245368999999 -4.395187360999999 6.839290683999999
F 3.655387387 -3.674565386 9.2024025
O 5.080874688 -1.718317566 1.698901385
O 5.112727950999999 -3.302516944 3.276859219
C 7.135493120999999 -2.576308034999999 2.548373772
H 0.8774352939 -5.538171384 -0.4179407209
H -0.8861899740999999 -5.543261926 -0.4179226517999999
H -0.004675634316 -5.432278195 1.017193679
H -5.339388166 0.008185713854999999 -1.022182427
H 0.8674911352999999 0.4407325033999999 -5.434834666
H -5.430458905999999 -0.8736724881 0.4319115204
H -0.01380144482 -1.013616356 -5.342369757
H -5.431324795 0.8899599141999999 0.4319079731
H -0.8961103623999999 0.4407446211 -5.424358162
H -2.502845664838624 5.776779325405495 -4.07758863532808
H -2.478721873199239 6.161353552997004 -4.500264932432724
C 1.51326616858716 5.511134480060788 -3.03687333538412
O 0.6925468896222865 4.945677675224854 -3.7083824903268
O 2.318608579592808 6.083236239459251 -2.393402202460863

FigureS5_a5

94
Frame 13: Geometry 14, SCF Bond Energy: -22.33301 Hartree
Zn 1.118325877 -1.128235255 1.117020731
O 0.0 0.0 0.0
Zn -1.116900352 1.12047199 1.123505959
Zn 1.122193945 1.118972622 -1.119718947
Zn -1.126215951 -1.116505389 -1.12339591
O 0.8021369693 -0.8117837309 3.008750804
C 0.003054830187 -0.008432567086999999 3.581919254
O -0.7970818184999999 0.7970313188999999 3.013234213
C 3.581912359 -0.01082908697 -0.003540432186
O 3.009680999 -0.8132619803999999 0.7971343812999999
O 3.012291901999999 0.7944804435999999 -0.8031669120999999
C 0.009180282035000001 3.581383465 0.006769578978999999
O 0.8084220440999999 3.010074891 -0.7966145626999999
O -3.009020291 0.8078659864999999 0.8060820827999999
C -0.003346512798000001 0.006652391428999999 -3.583579729000001
O -0.8071460657 -0.7947157234999999 -3.014216963
O 0.8010740201999999 0.8053146168000001 -3.011040958
C -3.583574564 0.009049827377000001 0.003253003893
O -3.016230165 -0.7932364457 -0.8010576277999999
O -0.7931109439999999 3.011147329 0.8078728793999999
C -0.01001744098 -3.585239577 -0.00760262035
O -0.8109746898 -3.011603871 -0.8074684318999998
O 0.7938278576999999 -3.018133795999999 0.794383446
C -0.005714743448 -5.075896192 -0.002311488867
H 8.944978446999999 -1.73802636 1.708825535
C 7.166557128 0.8509425830999998 -0.8656405017999999
B 7.797186308999999 -3.662463194 3.628658441999999
C -4.98356264 0.008362464348999999 -0.002482391202
C 5.736011636999999 -2.560830268 2.536480292999999
C 5.777265182999999 -0.8601664526 0.8423267605
H 7.727576396999999 1.515452898 -1.528974018
H 5.229417135 1.525289674 -1.532493864
C 5.776125075 0.8532885278 -0.8644797624
F 6.844399095999999 -4.396842793999999 4.362576411
F 9.206666662 -3.678282702 3.640882422
C -0.01166293599 0.005033113740999999 -4.983554086999999
C 5.075892805999999 -0.003126622774 -0.009635751917999999
H 8.957321327999999 -0.01270862644 -0.009821881387999998
C 7.167772842999999 -0.8600874402999998 0.8387531465999999
C 7.851202703 -1.725742503 1.699304045
C 7.863449254999999 -0.005265627748 -0.01449109999
C 0.04396635773 5.194640869999999 0.04041330098999999
C -0.8503016577999998 5.774818662 -0.8525958258999999

C -0.8472364843 7.165257035999999 -0.8504656691999998
C 0.0084575933 7.859690976999999 0.003188601708999999
C 0.8623193297 7.161415834000001 0.8559447949
C 0.8614153359999999 5.771067636999998 0.8559717714999999
O 1.718340578 5.072215416 1.711786215
C 2.560315049 5.724962876 2.551773997999999
C 2.576296403 7.12441826 2.566790525999999
C 1.727159976 7.842559230999999 1.718737673
O 3.300925372 5.099554367 3.291438609
B 3.661876094 7.782988681000001 3.649931079999999
F 4.395013939 6.828095789999999 4.382357207
F 3.678399509 9.192428536999998 3.665482008
H -1.52187739 5.230034086 -1.522570815
H -1.510781707 7.728179841999999 -1.513165323
H 0.01644550436 8.95356 0.01043066254
H 1.739992534 8.936287349000001 1.730815553
C 1.712619317 -1.721631909 7.849211683999999
C 0.0 0.0 7.863464373999999
C 2.559799639 -2.573243995 7.132508334999999
C 0.8513889166999999 -0.855857778 7.166789323999999
C -0.8518600472 0.8563119774999999 7.167568769999999
C 2.545336882 -2.558693685000001 5.733041101
C 0.8523993626999999 -0.856868599 5.7762778
C -0.8532607063 0.8577277862999999 5.777135683999999
C -0.0002814954319999999 0.000272557141 5.075902906999999
O 1.707115111 -1.71605838 5.078884757999999
O 3.284069746 -3.301292418 5.108890862
H 1.72414872 -1.733191061 8.942975999
H 0.006680556007 -0.006714976792999999 8.957330720999998
B 3.640575938 -3.659679796 7.792936794999999
H -1.513714005 1.521641171 7.729364400999998
H -1.521832107 1.529810538 5.231209839
F 4.372245368999999 -4.395187360999999 6.839290683999999
F 3.655387387 -3.674565386 9.2024025
O 5.080874688 -1.718317566 1.698901385
O 5.112727950999999 -3.302516944 3.276859219
C 7.135493120999999 -2.576308034999999 2.548373772
H 0.8774352939 -5.538171384 -0.4179407209
H -0.8861899740999999 -5.543261926 -0.4179226517999999
H -0.004675634316 -5.432278195 1.017193679
H -5.339388166 0.008185713854999999 -1.022182427
H 0.8674911352999999 0.4407325033999999 -5.434834666
H -5.430458905999999 -0.8736724881 0.4319115204
H -0.01380144482 -1.013616356 -5.342369757
H -5.431324795 0.8899599141999999 0.4319079731
H -0.8961103623999999 0.4407446211 -5.424358162
H -3.071151473143219 5.957431678105905 -4.190593584363062
H -2.293818737918141 6.243639072921872 -4.831597416818851

C 1.479730380324086 5.406742667038799 -3.059293126008745
O 0.7488983791306552 4.897672455248896 -3.740303414154308
O 2.265658834445987 5.929472478646962 -2.414988602415774

FigureS5_a6

94
Frame 17: Geometry 18, SCF Bond Energy: -22.34971 Hartree
Zn 1.118325877 -1.128235255 1.117020731
O 0.0 0.0 0.0
Zn -1.116900352 1.12047199 1.123505959
Zn 1.122193945 1.118972622 -1.119718947
Zn -1.126215951 -1.116505389 -1.12339591
O 0.8021369693 -0.8117837309 3.008750804
C 0.003054830187 -0.008432567086999999 3.581919254
O -0.7970818184999999 0.7970313188999999 3.013234213
C 3.581912359 -0.01082908697 -0.003540432186
O 3.009680999 -0.8132619803999999 0.7971343812999999
O 3.012291901999999 0.7944804435999999 -0.8031669120999999
C 0.009180282035000001 3.581383465 0.006769578978999999
O 0.8084220440999999 3.010074891 -0.7966145626999999
O -3.009020291 0.8078659864999999 0.8060820827999999
C -0.003346512798000001 0.006652391428999999 -3.583579729000001
O -0.8071460657 -0.7947157234999999 -3.014216963
O 0.8010740201999999 0.8053146168000001 -3.011040958
C -3.583574564 0.009049827377000001 0.003253003893
O -3.016230165 -0.7932364457 -0.8010576277999999
O -0.7931109439999999 3.011147329 0.8078728793999999
C -0.01001744098 -3.585239577 -0.00760262035
O -0.8109746898 -3.011603871 -0.8074684318999998
O 0.7938278576999999 -3.018133795999999 0.794383446
C -0.005714743448 -5.075896192 -0.002311488867
H 8.944978446999999 -1.73802636 1.708825535
C 7.166557128 0.8509425830999998 -0.8656405017999999
B 7.797186308999999 -3.662463194 3.628658441999999
C -4.98356264 0.008362464348999999 -0.002482391202
C 5.736011636999999 -2.560830268 2.536480292999999
C 5.777265182999999 -0.8601664526 0.8423267605
H 7.727576396999999 1.515452898 -1.528974018
H 5.229417135 1.525289674 -1.532493864
C 5.776125075 0.8532885278 -0.8644797624
F 6.844399095999999 -4.396842793999999 4.362576411
F 9.206666662 -3.678282702 3.640882422
C -0.01166293599 0.005033113740999999 -4.983554086999999
C 5.075892805999999 -0.003126622774 -0.009635751917999999
H 8.957321327999999 -0.01270862644 -0.009821881387999998

C 7.167772842999999 -0.8600874402999998 0.8387531465999999
C 7.851202703 -1.725742503 1.699304045
C 7.863449254999999 -0.005265627748 -0.01449109999
C 0.04396635773 5.194640869999999 0.04041330098999999
C -0.8503016577999998 5.774818662 -0.8525958258999999
C -0.8472364843 7.165257035999999 -0.8504656691999998
C 0.0084575933 7.859690976999999 0.003188601708999999
C 0.8623193297 7.161415834000001 0.8559447949
C 0.8614153359999999 5.771067636999998 0.8559717714999999
O 1.718340578 5.072215416 1.711786215
C 2.560315049 5.724962876 2.551773997999999
C 2.576296403 7.12441826 2.566790525999999
C 1.727159976 7.842559230999999 1.718737673
O 3.300925372 5.099554367 3.291438609
B 3.661876094 7.782988681000001 3.649931079999999
F 4.395013939 6.828095789999999 4.382357207
F 3.678399509 9.192428536999998 3.665482008
H -1.52187739 5.230034086 -1.522570815
H -1.510781707 7.728179841999999 -1.513165323
H 0.01644550436 8.95356 0.01043066254
H 1.739992534 8.936287349000001 1.730815553
C 1.712619317 -1.721631909 7.849211683999999
C 0.0 0.0 7.863464373999999
C 2.559799639 -2.573243995 7.132508334999999
C 0.8513889166999999 -0.855857778 7.166789323999999
C -0.8518600472 0.8563119774999999 7.167568769999999
C 2.545336882 -2.558693685000001 5.733041101
C 0.8523993626999999 -0.856868599 5.7762778
C -0.8532607063 0.8577277862999999 5.777135683999999
C -0.0002814954319999999 0.000272557141 5.075902906999999
O 1.707115111 -1.71605838 5.078884757999999
O 3.284069746 -3.301292418 5.108890862
H 1.72414872 -1.733191061 8.942975999
H 0.006680556007 -0.006714976792999999 8.957330720999998
B 3.640575938 -3.659679796 7.792936794999999
H -1.513714005 1.521641171 7.729364400999998
H -1.521832107 1.529810538 5.231209839
F 4.372245368999999 -4.395187360999999 6.839290683999999
F 3.655387387 -3.674565386 9.2024025
O 5.080874688 -1.718317566 1.698901385
O 5.112727950999999 -3.302516944 3.276859219
C 7.135493120999999 -2.576308034999999 2.548373772
H 0.8774352939 -5.538171384 -0.4179407209
H -0.8861899740999999 -5.543261926 -0.4179226517999999
H -0.004675634316 -5.432278195 1.017193679
H -5.339388166 0.008185713854999999 -1.022182427
H 0.8674911352999999 0.4407325033999999 -5.434834666
H -5.430458905999999 -0.8736724881 0.4319115204

H -0.01380144482 -1.013616356 -5.342369757
H -5.431324795 0.8899599141999999 0.4319079731
H -0.8961103623999999 0.4407446211 -5.424358162
H -3.072233639005895 5.845567227202834 -4.300493355818586
H -2.501876102665909 6.3919862208118 -4.88302703199054
C 1.501802818687155 5.384100498702789 -3.078137999390074
O 0.6483139124326297 4.903248474336331 -3.738852946335403
O 2.379308200458677 5.850615327650377 -2.43612689858329

FigureS6_b1

95
Frame 1: Geometry 2, SCF Bond Energy: -22.55634 Hartree
Zn 1.589227043 -1.29697559 1.416164534
O 0.7365071251999998 -0.7780997006999999 -0.2496504088
Zn -0.8820363213999999 0.2207960636 0.1346852258
Zn 1.951109251 0.328212789 -1.282125033
Zn 0.2871873256999999 -2.37065937 -1.268880726
O 0.5913578901999999 -0.672892423 2.963270548
C -0.4878202884999999 -0.006751519597000001 3.026974431999999
O -1.176673843 0.4129351586 2.046292386
C 4.040539404 0.1649380559 0.762415026
O 3.384582631 -0.5669892729999999 1.566424064
O 3.643359998 0.5956833602999999 -0.3641285439999999
C 0.09092655746 2.588954508 -1.285318945
O 1.206995178 2.090725852 -1.627029351
O -2.459916077 -0.6456766368 -0.5988806059999999
C 1.961459269 -1.551558128 -3.527355922
O 1.171256328 -2.394430197 -3.000474033
O 2.361029534 -0.4628938763 -3.00975749
C -2.568634555 -1.723313455 -1.261929307
O -1.623917076 -2.500407231 -1.602653051
O -0.8194385007 2.013895043 -0.6136426593999999
C 1.382303687 -4.149179039 0.7861448729
O 0.8141806916 -3.997653924 -0.3384226684
O 1.744775623 -3.2282482 1.58078842
C 1.65571441 -5.546700859999999 1.226920317
H 8.709702646 0.3434940855999999 4.346395753000001
C 7.482803937000001 1.741539476 0.7361870891
B 7.343627763999998 -1.352128542 6.337713313999999
C -3.856676059 -2.098066428 -1.662565392
C 5.618361794 -1.10394527 4.435937397999999
C 5.928033708999999 0.1358812153 2.403439169
H 8.106415634999999 2.370811123 0.09474061705999999
H 5.803699031 1.713944798 -0.6172507577999999
C 6.200651105999999 1.373678762 0.3436686076

F 6.347491698 -2.136011877 6.953336827999999
F 8.641194798999999 -0.9889382821 6.751775113
C 2.434312064999999 -1.859548038 -4.808586752
C 5.417979525 0.5687406225 1.176753363
H 8.996648026000001 1.593449064 2.276154832
C 7.210642604 0.5055148625999999 2.793066834999999
C 7.702783926 0.06165306763 4.025043514
C 7.988857009 1.308091066 1.960621328
C -0.1821282627 4.120478193 -1.715223227
C -0.8054804096999999 4.233052423 -2.953183646
C -1.056409962 5.540038343999999 -3.355868347
C -0.6870514462 6.605576050999999 -2.536490444
C -0.06563670378 6.362591610000001 -1.31229134
C 0.186528091 5.056734768 -0.9070509766999999
O 0.8098219182999999 4.814688969 0.3209768304
C 1.178811134 5.834456441 1.136146135
C 0.9337230876 7.156150446999999 0.746257596
C 0.3114344579 7.420070784 -0.4781016994999999
O 1.721394136 5.605157766999999 2.203745777
B 1.442735961 8.299109833999999 1.850767198
F 2.040873357 7.7568419 3.005728976
F 1.196147217 9.630440378999998 1.458608386
H -1.095406273 3.396999896 -3.596324261
H -1.543004904 5.747910674 -4.3131351
H -0.8812301468 7.636487265999999 -2.846623306
H 0.1200871037 8.453190924999998 -0.7824053487
C -0.06441925129999999 -0.2328349847 7.909296136999999
C -1.960834017 0.9308555253999999 6.936300799
C 1.117100802 -0.9626800089999998 7.742089861999999
C -0.7815331501999999 0.2024971394 6.7898246
C -2.66296935 1.356869609 5.809924126999999
C 1.581173155 -1.257012437 6.454870108999999
C -0.3034420286 -0.100380808 5.519699341999999
C -2.187568628999999 1.055645309 4.538481556999999
C -1.00494816 0.3252198403 4.388755562
O 0.8797772447 -0.8311381482999999 5.374478798999999
O 2.611170056 -1.89325581 6.309806681
H -0.4268762763 -0.002912890016 8.915451022999999
H -2.328675923 1.16405737 7.939766063999999
B 2.084232353 -1.551534826 8.96785186
H -3.586669828999999 1.927476006 5.942263439
H -2.738715601 1.390065152 3.654672553999999
F 3.219453994 -2.25460923 8.517617866999999
F 1.617112647 -1.255251425 10.26440493
O 5.148624706999999 -0.6692878018999999 3.239697265
O 4.924987251999999 -1.805529052 5.152704147999999
C 6.906765731999999 -0.7431689363 4.846772123
C 3.694326815079481 -4.042690870403033 3.242238753925445

O 4.860326800909784 -3.735934475782102 2.770304076879926
O 3.364471566688087 -4.47959505111999 4.414686881419326
H -3.834769012 -2.414609901 -2.694902696999999
H 3.319993996999999 -1.314329337 -5.099626409999999
H -4.257004619999999 -2.950967556 -1.13464806
H 2.740790656999999 -2.894672593 -4.840019667999999
H -4.578736444 -1.295040313 -1.649229052
H 1.691182728 -1.781743883 -5.588397819999999
H 2.69625751 -5.835922873000001 1.231468292
H 1.07194036 -6.310408376 0.7346209281999999
H 1.371811528 -5.659521028 2.262811605
O 4.261084012043564 -2.586912085682901 -0.3827260184175121
H 4.322642751767124 -2.986053963584021 0.5338979302706692
H 4.801965404660738 -3.235336214150384 -0.9182178996159039

FigureS6_b2

95
Frame 5: Geometry 6, SCF Bond Energy: -22.58520 Hartree
Zn 1.589227043 -1.29697559 1.416164534
O 0.7365071251999998 -0.7780997006999999 -0.2496504088
Zn -0.8820363213999999 0.2207960636 0.1346852258
Zn 1.951109251 0.328212789 -1.282125033
Zn 0.2871873256999999 -2.37065937 -1.268880726
O 0.5913578901999999 -0.672892423 2.963270548
C -0.4878202884999999 -0.006751519597000001 3.026974431999999
O -1.176673843 0.4129351586 2.046292386
C 4.040539404 0.1649380559 0.762415026
O 3.384582631 -0.5669892729999999 1.566424064
O 3.643359998 0.5956833602999999 -0.3641285439999999
C 0.09092655746 2.588954508 -1.285318945
O 1.206995178 2.090725852 -1.627029351
O -2.459916077 -0.6456766368 -0.5988806059999999
C 1.961459269 -1.551558128 -3.527355922
O 1.171256328 -2.394430197 -3.000474033
O 2.361029534 -0.4628938763 -3.00975749
C -2.568634555 -1.723313455 -1.261929307
O -1.623917076 -2.500407231 -1.602653051
O -0.8194385007 2.013895043 -0.6136426593999999
C 1.382303687 -4.149179039 0.7861448729
O 0.8141806916 -3.997653924 -0.3384226684
O 1.744775623 -3.2282482 1.58078842
C 1.65571441 -5.546700859999999 1.226920317
H 8.709702646 0.3434940855999999 4.346395753000001
C 7.482803937000001 1.741539476 0.7361870891
B 7.343627763999998 -1.352128542 6.337713313999999

C -3.856676059 -2.098066428 -1.662565392
C 5.618361794 -1.10394527 4.435937397999999
C 5.928033708999999 0.1358812153 2.403439169
H 8.106415634999999 2.370811123 0.09474061705999999
H 5.803699031 1.713944798 -0.6172507577999999
C 6.200651105999999 1.373678762 0.3436686076
F 6.347491698 -2.136011877 6.953336827999999
F 8.641194798999999 -0.9889382821 6.751775113
C 2.434312064999999 -1.859548038 -4.808586752
C 5.417979525 0.5687406225 1.176753363
H 8.996648026000001 1.593449064 2.276154832
C 7.210642604 0.5055148625999999 2.793066834999999
C 7.702783926 0.06165306763 4.025043514
C 7.988857009 1.308091066 1.960621328
C -0.1821282627 4.120478193 -1.715223227
C -0.8054804096999999 4.233052423 -2.953183646
C -1.056409962 5.540038343999999 -3.355868347
C -0.6870514462 6.605576050999999 -2.536490444
C -0.06563670378 6.362591610000001 -1.31229134
C 0.186528091 5.056734768 -0.9070509766999999
O 0.8098219182999999 4.814688969 0.3209768304
C 1.178811134 5.834456441 1.136146135
C 0.9337230876 7.156150446999999 0.746257596
C 0.3114344579 7.420070784 -0.4781016994999999
O 1.721394136 5.605157766999999 2.203745777
B 1.442735961 8.299109833999999 1.850767198
F 2.040873357 7.7568419 3.005728976
F 1.196147217 9.630440378999998 1.458608386
H -1.095406273 3.396999896 -3.596324261
H -1.543004904 5.747910674 -4.3131351
H -0.8812301468 7.636487265999999 -2.846623306
H 0.1200871037 8.453190924999998 -0.7824053487
C -0.06441925129999999 -0.2328349847 7.909296136999999
C -1.960834017 0.9308555253999999 6.936300799
C 1.117100802 -0.9626800089999998 7.742089861999999
C -0.7815331501999999 0.2024971394 6.7898246
C -2.66296935 1.356869609 5.809924126999999
C 1.581173155 -1.257012437 6.454870108999999
C -0.3034420286 -0.100380808 5.519699341999999
C -2.187568628999999 1.055645309 4.538481556999999
C -1.00494816 0.3252198403 4.388755562
O 0.8797772447 -0.8311381482999999 5.374478798999999
O 2.611170056 -1.89325581 6.309806681
H -0.4268762763 -0.002912890016 8.915451022999999
H -2.328675923 1.16405737 7.939766063999999
B 2.084232353 -1.551534826 8.96785186
H -3.586669828999999 1.927476006 5.942263439
H -2.738715601 1.390065152 3.654672553999999

F 3.219453994 -2.25460923 8.517617866999999
F 1.617112647 -1.255251425 10.26440493
O 5.148624706999999 -0.6692878018999999 3.239697265
O 4.924987251999999 -1.805529052 5.152704147999999
C 6.906765731999999 -0.7431689363 4.846772123
C 3.894749516841932 -4.257429571592179 3.311231137885951
O 4.84064160441522 -3.889236914186391 2.699588689803462
O 3.551886343865807 -4.6895558292304 4.412031927648129
H -3.834769012 -2.414609901 -2.694902696999999
H 3.319993996999999 -1.314329337 -5.099626409999999
H -4.257004619999999 -2.950967556 -1.13464806
H 2.740790656999999 -2.894672593 -4.840019667999999
H -4.578736444 -1.295040313 -1.649229052
H 1.691182728 -1.781743883 -5.588397819999999
H 2.69625751 -5.835922873000001 1.231468292
H 1.07194036 -6.310408376 0.7346209281999999
H 1.371811528 -5.659521028 2.262811605
O 4.209605499977926 -2.815347719844498 -0.3632402935821819
H 4.316179263050549 -2.925867703628438 0.5118091152868062
H 4.833647591672182 -3.131765751397182 -0.8770400165769199

FigureS6_b3

95
Frame 18: Geometry 19, SCF Bond Energy: -22.62203 Hartree
Zn 1.589227043 -1.29697559 1.416164534
O 0.7365071251999998 -0.7780997006999999 -0.2496504088
Zn -0.8820363213999999 0.2207960636 0.1346852258
Zn 1.951109251 0.328212789 -1.282125033
Zn 0.2871873256999999 -2.37065937 -1.268880726
O 0.5913578901999999 -0.672892423 2.963270548
C -0.4878202884999999 -0.006751519597000001 3.026974431999999
O -1.176673843 0.4129351586 2.046292386
C 4.040539404 0.1649380559 0.762415026
O 3.384582631 -0.5669892729999999 1.566424064
O 3.643359998 0.5956833602999999 -0.3641285439999999
C 0.09092655746 2.588954508 -1.285318945
O 1.206995178 2.090725852 -1.627029351
O -2.459916077 -0.6456766368 -0.5988806059999999
C 1.961459269 -1.551558128 -3.527355922
O 1.171256328 -2.394430197 -3.000474033
O 2.361029534 -0.4628938763 -3.00975749
C -2.568634555 -1.723313455 -1.261929307
O -1.623917076 -2.500407231 -1.602653051
O -0.8194385007 2.013895043 -0.6136426593999999
C 1.382303687 -4.149179039 0.7861448729

O 0.8141806916 -3.997653924 -0.3384226684
O 1.744775623 -3.2282482 1.58078842
C 1.65571441 -5.546700859999999 1.226920317
H 8.709702646 0.3434940855999999 4.346395753000001
C 7.482803937000001 1.741539476 0.7361870891
B 7.343627763999998 -1.352128542 6.337713313999999
C -3.856676059 -2.098066428 -1.662565392
C 5.618361794 -1.10394527 4.435937397999999
C 5.928033708999999 0.1358812153 2.403439169
H 8.106415634999999 2.370811123 0.09474061705999999
H 5.803699031 1.713944798 -0.6172507577999999
C 6.200651105999999 1.373678762 0.3436686076
F 6.347491698 -2.136011877 6.953336827999999
F 8.641194798999999 -0.9889382821 6.751775113
C 2.434312064999999 -1.859548038 -4.808586752
C 5.417979525 0.5687406225 1.176753363
H 8.996648026000001 1.593449064 2.276154832
C 7.210642604 0.5055148625999999 2.793066834999999
C 7.702783926 0.06165306763 4.025043514
C 7.988857009 1.308091066 1.960621328
C -0.1821282627 4.120478193 -1.715223227
C -0.8054804096999999 4.233052423 -2.953183646
C -1.056409962 5.540038343999999 -3.355868347
C -0.6870514462 6.605576050999999 -2.536490444
C -0.06563670378 6.362591610000001 -1.31229134
C 0.186528091 5.056734768 -0.9070509766999999
O 0.8098219182999999 4.814688969 0.3209768304
C 1.178811134 5.834456441 1.136146135
C 0.9337230876 7.156150446999999 0.746257596
C 0.3114344579 7.420070784 -0.4781016994999999
O 1.721394136 5.605157766999999 2.203745777
B 1.442735961 8.299109833999999 1.850767198
F 2.040873357 7.7568419 3.005728976
F 1.196147217 9.630440378999998 1.458608386
H -1.095406273 3.396999896 -3.596324261
H -1.543004904 5.747910674 -4.3131351
H -0.8812301468 7.636487265999999 -2.846623306
H 0.1200871037 8.453190924999998 -0.7824053487
C -0.06441925129999999 -0.2328349847 7.909296136999999
C -1.960834017 0.9308555253999999 6.936300799
C 1.117100802 -0.9626800089999998 7.742089861999999
C -0.7815331501999999 0.2024971394 6.7898246
C -2.66296935 1.356869609 5.809924126999999
C 1.581173155 -1.257012437 6.454870108999999
C -0.3034420286 -0.100380808 5.519699341999999
C -2.187568628999999 1.055645309 4.538481556999999
C -1.00494816 0.3252198403 4.388755562
O 0.8797772447 -0.8311381482999999 5.374478798999999

O 2.611170056 -1.89325581 6.309806681
H -0.4268762763 -0.002912890016 8.915451022999999
H -2.328675923 1.16405737 7.939766063999999
B 2.084232353 -1.551534826 8.96785186
H -3.586669828999999 1.927476006 5.942263439
H -2.738715601 1.390065152 3.654672553999999
F 3.219453994 -2.25460923 8.517617866999999
F 1.617112647 -1.255251425 10.26440493
O 5.148624706999999 -0.6692878018999999 3.239697265
O 4.924987251999999 -1.805529052 5.152704147999999
C 6.906765731999999 -0.7431689363 4.846772123
C 3.926426084116205 -4.187615944944578 3.483292575096194
O 4.84106798147389 -3.607547872876708 2.844075175015749
O 2.972656186239097 -4.753000609926853 4.040228343180913
H -3.834769012 -2.414609901 -2.694902696999999
H 3.319993996999999 -1.314329337 -5.099626409999999
H -4.257004619999999 -2.950967556 -1.13464806
H 2.740790656999999 -2.894672593 -4.840019667999999
H -4.578736444 -1.295040313 -1.649229052
H 1.691182728 -1.781743883 -5.588397819999999
H 2.69625751 -5.835922873000001 1.231468292
H 1.07194036 -6.310408376 0.7346209281999999
H 1.371811528 -5.659521028 2.262811605
O 4.152418991391459 -2.764299451662027 -0.4007821576207439
H 4.225914595067615 -2.970746758182306 0.5983588414171982
H 4.767797255454278 -3.402010131511296 -0.9005386900301813

FigureS6_b4

95
Frame 20: Geometry 21, SCF Bond Energy: -22.60372 Hartree
Zn 1.589227043 -1.29697559 1.416164534
O 0.7365071251999998 -0.7780997006999999 -0.2496504088
Zn -0.8820363213999999 0.2207960636 0.1346852258
Zn 1.951109251 0.328212789 -1.282125033
Zn 0.2871873256999999 -2.37065937 -1.268880726
O 0.5913578901999999 -0.672892423 2.963270548
C -0.4878202884999999 -0.006751519597000001 3.026974431999999
O -1.176673843 0.4129351586 2.046292386
C 4.040539404 0.1649380559 0.762415026
O 3.384582631 -0.5669892729999999 1.566424064
O 3.643359998 0.5956833602999999 -0.3641285439999999
C 0.09092655746 2.588954508 -1.285318945
O 1.206995178 2.090725852 -1.627029351
O -2.459916077 -0.6456766368 -0.5988806059999999
C 1.961459269 -1.551558128 -3.527355922

O 1.171256328 -2.394430197 -3.000474033
O 2.361029534 -0.4628938763 -3.00975749
C -2.568634555 -1.723313455 -1.261929307
O -1.623917076 -2.500407231 -1.602653051
O -0.8194385007 2.013895043 -0.6136426593999999
C 1.382303687 -4.149179039 0.7861448729
O 0.8141806916 -3.997653924 -0.3384226684
O 1.744775623 -3.2282482 1.58078842
C 1.65571441 -5.546700859999999 1.226920317
H 8.709702646 0.3434940855999999 4.346395753000001
C 7.482803937000001 1.741539476 0.7361870891
B 7.343627763999998 -1.352128542 6.337713313999999
C -3.856676059 -2.098066428 -1.662565392
C 5.618361794 -1.10394527 4.435937397999999
C 5.928033708999999 0.1358812153 2.403439169
H 8.106415634999999 2.370811123 0.09474061705999999
H 5.803699031 1.713944798 -0.6172507577999999
C 6.200651105999999 1.373678762 0.3436686076
F 6.347491698 -2.136011877 6.953336827999999
F 8.641194798999999 -0.9889382821 6.751775113
C 2.434312064999999 -1.859548038 -4.808586752
C 5.417979525 0.5687406225 1.176753363
H 8.996648026000001 1.593449064 2.276154832
C 7.210642604 0.5055148625999999 2.793066834999999
C 7.702783926 0.06165306763 4.025043514
C 7.988857009 1.308091066 1.960621328
C -0.1821282627 4.120478193 -1.715223227
C -0.8054804096999999 4.233052423 -2.953183646
C -1.056409962 5.540038343999999 -3.355868347
C -0.6870514462 6.605576050999999 -2.536490444
C -0.06563670378 6.362591610000001 -1.31229134
C 0.186528091 5.056734768 -0.9070509766999999
O 0.8098219182999999 4.814688969 0.3209768304
C 1.178811134 5.834456441 1.136146135
C 0.9337230876 7.156150446999999 0.746257596
C 0.3114344579 7.420070784 -0.4781016994999999
O 1.721394136 5.605157766999999 2.203745777
B 1.442735961 8.299109833999999 1.850767198
F 2.040873357 7.7568419 3.005728976
F 1.196147217 9.630440378999998 1.458608386
H -1.095406273 3.396999896 -3.596324261
H -1.543004904 5.747910674 -4.3131351
H -0.8812301468 7.636487265999999 -2.846623306
H 0.1200871037 8.453190924999998 -0.7824053487
C -0.06441925129999999 -0.2328349847 7.909296136999999
C -1.960834017 0.9308555253999999 6.936300799
C 1.117100802 -0.9626800089999998 7.742089861999999
C -0.7815331501999999 0.2024971394 6.7898246

C -2.66296935 1.356869609 5.809924126999999
C 1.581173155 -1.257012437 6.454870108999999
C -0.3034420286 -0.100380808 5.519699341999999
C -2.187568628999999 1.055645309 4.538481556999999
C -1.00494816 0.3252198403 4.388755562
O 0.8797772447 -0.8311381482999999 5.374478798999999
O 2.611170056 -1.89325581 6.309806681
H -0.4268762763 -0.002912890016 8.915451022999999
H -2.328675923 1.16405737 7.939766063999999
B 2.084232353 -1.551534826 8.96785186
H -3.586669828999999 1.927476006 5.942263439
H -2.738715601 1.390065152 3.654672553999999
F 3.219453994 -2.25460923 8.517617866999999
F 1.617112647 -1.255251425 10.26440493
O 5.148624706999999 -0.6692878018999999 3.239697265
O 4.924987251999999 -1.805529052 5.152704147999999
C 6.906765731999999 -0.7431689363 4.846772123
C 3.930406976794207 -4.210467312815887 3.424670582793679
O 4.788409708711532 -3.862169979025371 2.844965901449463
O 3.032767179344934 -4.569385170587069 4.090575810969984
H -3.834769012 -2.414609901 -2.694902696999999
H 3.319993996999999 -1.314329337 -5.099626409999999
H -4.257004619999999 -2.950967556 -1.13464806
H 2.740790656999999 -2.894672593 -4.840019667999999
H -4.578736444 -1.295040313 -1.649229052
H 1.691182728 -1.781743883 -5.588397819999999
H 2.69625751 -5.835922873000001 1.231468292
H 1.07194036 -6.310408376 0.7346209281999999
H 1.371811528 -5.659521028 2.262811605
O 3.998983273374593 -2.821911758298496 -0.3237803704505314
H 4.486461335214043 -3.048340017648728 0.4639834501087399
H 4.589031831519832 -3.184478307584324 -0.8113188949592239

FigureS6_b5

95
Frame 61: Geometry 62, SCF Bond Energy: -22.62613 Hartree
Zn 1.589227043 -1.29697559 1.416164534
O 0.7365071251999998 -0.7780997006999999 -0.2496504088
Zn -0.8820363213999999 0.2207960636 0.1346852258
Zn 1.951109251 0.328212789 -1.282125033
Zn 0.2871873256999999 -2.37065937 -1.268880726
O 0.5913578901999999 -0.672892423 2.963270548
C -0.4878202884999999 -0.006751519597000001 3.026974431999999
O -1.176673843 0.4129351586 2.046292386
C 4.040539404 0.1649380559 0.762415026

O 3.384582631 -0.5669892729999999 1.566424064
O 3.643359998 0.5956833602999999 -0.3641285439999999
C 0.09092655746 2.588954508 -1.285318945
O 1.206995178 2.090725852 -1.627029351
O -2.459916077 -0.6456766368 -0.5988806059999999
C 1.961459269 -1.551558128 -3.527355922
O 1.171256328 -2.394430197 -3.000474033
O 2.361029534 -0.4628938763 -3.00975749
C -2.568634555 -1.723313455 -1.261929307
O -1.623917076 -2.500407231 -1.602653051
O -0.8194385007 2.013895043 -0.6136426593999999
C 1.382303687 -4.149179039 0.7861448729
O 0.8141806916 -3.997653924 -0.3384226684
O 1.744775623 -3.2282482 1.58078842
C 1.65571441 -5.546700859999999 1.226920317
H 8.709702646 0.3434940855999999 4.346395753000001
C 7.482803937000001 1.741539476 0.7361870891
B 7.343627763999998 -1.352128542 6.337713313999999
C -3.856676059 -2.098066428 -1.662565392
C 5.618361794 -1.10394527 4.435937397999999
C 5.928033708999999 0.1358812153 2.403439169
H 8.106415634999999 2.370811123 0.09474061705999999
H 5.803699031 1.713944798 -0.6172507577999999
C 6.200651105999999 1.373678762 0.3436686076
F 6.347491698 -2.136011877 6.953336827999999
F 8.641194798999999 -0.9889382821 6.751775113
C 2.434312064999999 -1.859548038 -4.808586752
C 5.417979525 0.5687406225 1.176753363
H 8.996648026000001 1.593449064 2.276154832
C 7.210642604 0.5055148625999999 2.793066834999999
C 7.702783926 0.06165306763 4.025043514
C 7.988857009 1.308091066 1.960621328
C -0.1821282627 4.120478193 -1.715223227
C -0.8054804096999999 4.233052423 -2.953183646
C -1.056409962 5.540038343999999 -3.355868347
C -0.6870514462 6.605576050999999 -2.536490444
C -0.06563670378 6.362591610000001 -1.31229134
C 0.186528091 5.056734768 -0.9070509766999999
O 0.8098219182999999 4.814688969 0.3209768304
C 1.178811134 5.834456441 1.136146135
C 0.9337230876 7.156150446999999 0.746257596
C 0.3114344579 7.420070784 -0.4781016994999999
O 1.721394136 5.605157766999999 2.203745777
B 1.442735961 8.299109833999999 1.850767198
F 2.040873357 7.7568419 3.005728976
F 1.196147217 9.630440378999998 1.458608386
H -1.095406273 3.396999896 -3.596324261
H -1.543004904 5.747910674 -4.3131351

H -0.8812301468 7.636487265999999 -2.846623306
H 0.1200871037 8.453190924999998 -0.7824053487
C -0.06441925129999999 -0.2328349847 7.909296136999999
C -1.960834017 0.9308555253999999 6.936300799
C 1.117100802 -0.9626800089999998 7.742089861999999
C -0.7815331501999999 0.2024971394 6.7898246
C -2.66296935 1.356869609 5.809924126999999
C 1.581173155 -1.257012437 6.454870108999999
C -0.3034420286 -0.100380808 5.519699341999999
C -2.187568628999999 1.055645309 4.538481556999999
C -1.00494816 0.3252198403 4.388755562
O 0.8797772447 -0.8311381482999999 5.374478798999999
O 2.611170056 -1.89325581 6.309806681
H -0.4268762763 -0.002912890016 8.915451022999999
H -2.328675923 1.16405737 7.939766063999999
B 2.084232353 -1.551534826 8.96785186
H -3.586669828999999 1.927476006 5.942263439
H -2.738715601 1.390065152 3.654672553999999
F 3.219453994 -2.25460923 8.517617866999999
F 1.617112647 -1.255251425 10.26440493
O 5.148624706999999 -0.6692878018999999 3.239697265
O 4.924987251999999 -1.805529052 5.152704147999999
C 6.906765731999999 -0.7431689363 4.846772123
C 3.670839379900157 -4.355758094383274 3.600029294461301
O 4.592032067328109 -3.905161223950969 3.004765913181028
O 2.757122326059812 -4.803708826144915 4.18411464246047
H -3.834769012 -2.414609901 -2.694902696999999
H 3.319993996999999 -1.314329337 -5.099626409999999
H -4.257004619999999 -2.950967556 -1.13464806
H 2.740790656999999 -2.894672593 -4.840019667999999
H -4.578736444 -1.295040313 -1.649229052
H 1.691182728 -1.781743883 -5.588397819999999
H 2.69625751 -5.835922873000001 1.231468292
H 1.07194036 -6.310408376 0.7346209281999999
H 1.371811528 -5.659521028 2.262811605
O 5.781910920729237 -2.315318908055824 0.6008558988817467
H 5.103659076777527 -2.991187316529357 1.135255109545773
H 5.850251378491905 -3.006881723678024 -0.2182687567809575
